# Paleogenomic evidence for genetic heterogeneity and prior admixture in Gothic-associated communities of late antique Bulgaria

**DOI:** 10.3389/fgene.2026.1864752

**Published:** 2026-07-24

**Authors:** Svetoslav Stamov, Todor Chobanov, Tianyi Wang, Kremena Stoeva, Dimcho Momchilov, Andrey Aladzhov, Kaloyan Chobanov, Miroslav Klasnakov, Georgi Stamov, Milen Nikolov, Desislava Nesheva, Peter Heather, Draga I. Toncheva, Milen Zamfirov, Iosif Lazaridis, David Reich

**Affiliations:** 1 Laboratory of Interdisciplinary Research and Paleogenomic Knowledge, National Museum of History, Sofia, Bulgaria; 2 UNIBIT, Sofia, Bulgaria; 3 Institute for Balkan Studies with Centre for Thracology, Bulgarian Academy of Sciences, Sofia, Bulgaria; 4 Department of Human Evolutionary Biology, Harvard University, Cambridge, MA, United States; 5 Department of Genetics, Harvard Medical School, Boston, MA, United States; 6 Archaeological Museum, Veliki Preslav, Bulgaria; 7 Regional Historical Museum, Burgas, Bulgaria; 8 Asen Zlatarov University, Burgas, Bulgaria; 9 Department of Medieval Archaeology, National Archaeological Institute with Museum, Bulgarian Academy of Sciences, Sofia, Bulgaria; 10 Medical University of Sofia, Sofia, Bulgaria; 11 Department of History, King’s College London, London, United Kingdom; 12 Department of Biological Sciences, Bulgarian Academy of Sciences, Sofia, Bulgaria; 13 Faculty of Educational Studies and the Arts, Sofia University St. Kliment Ohridski, Sofia, Bulgaria; 14 Howard Hughes Medical Institute, Harvard Medical School, Boston, MA, United States; 15 Broad Institute of MIT and Harvard, Cambridge, MA, United States

**Keywords:** admixture, ancient DNA, Goths, late antique Bulgaria, late antiquity, migration period, paleogenomics

## Abstract

We report genome-wide ancient DNA from 37 individuals retained after conservative archaeological and genomic reassessment of 53 screened samples from two Gothic-period mortuary contexts in present-day Bulgaria: the Aquae Calidae necropolis in Thrace (n = 20; c. 320 CE–375 CE) and the Aul of Khan Omurtag (AKO) in Moesia Secunda (n = 17; c. 350 CE–489 CE). The filtering process excluded individuals with uncertain or probably later-medieval horizon assignment, including a C5-area component linked by READv2 kinship to suspected later-horizon burials. We evaluate three non-exclusive models for Balkan Gothic formation, namely, migration-continuity from northern and Pontic Gothic horizons, a Wenskus–Wolfram–Pohl tradition-bearing cultural–political formation model across changing demographic substrates, and the Roman frontier formation. The two assemblages share Gothic-associated material culture and Christian east–west burial orientation, but they are not a single genome-wide population. Proximal qpAdm models distinguish a southern Anatolian/Marmara-related and northern/Pontic structure at Aquae Calidae from a simpler Chernyakhov-related and Balkan Late Antique structure at AKO. f_4_ statistics further show that the Anatolian-related component at Aquae Calidae cannot be explained solely as a Chernyakhov-carried signal. DATES places north–south admixture at approximately 12.44 ± 2.36 generations before burial (Z = 5.26), consistent with a pre-burial ancestry mixture rather than a single intact Gothic migration. Kinship is confined within sites. Together, the data reject a single biological Gothic population moving unchanged into the Balkans and instead support a formation process combining migration, multiethnic coalition, and frontier incorporation.

## 1 Introduction

### 1.1 Gothic origins as a chain of hypotheses

The familiar historical sequence Scandinavia → lower Vistula/Wielbark → Chernyakhov–Sântana de Mureş → lower Danube and Balkans has long structured the discussion of Gothic origins. Yet, this sequence is not a single hypothesis. It is a chain of linked propositions, and the evidence supporting each link differs in kind and strength. Ancient DNA has made a substantial Scandinavian-related ancestry component in many Wielbark-associated burials much harder to dismiss (Stolarek et al., 2023; Golubiński et al., 2026; see Supplementary Note S1 for a study-by-study comparison). It has not, by itself, proved the full Jordanes narrative (Jordanes, 1915), nor has it shown that every community archaeologically called Gothic was biologically continuous from Scandinavia to the Balkans (Kulikowski, 2007; Goffart, 1988; historiographic background summarised in Supplementary Note S2).

The Wielbark horizon of northern Poland remains the strongest archaeogenomic anchor for a northern component in Gothic-associated population history (Stolarek et al., 2023). Published genome-wide studies model Wielbark-associated individuals as predominantly northern European or Scandinavian-related, while also showing admixture with local central and east-central European populations (Stolarek et al., 2023; Stolarek et al., 2019). The Masłomęcz group, a Wielbark-associated assemblage in eastern Poland, combines a strong Scandinavian-derived component with clear assimilation of Baltic, Balkan, western/central European, and Mediterranean-related individuals (Golubiński et al., 2026; Stolarek et al., 2019). Thus, even in the zone that is often treated as a Gothic core, the genomic signal is not one of biological purity but of mobility and incorporation (Golubiński et al., 2026).

The next link, the Chernyakhov–Sântana de Mureş horizon of present-day Ukraine, Moldova, and Romania, is more complex. Migrationist historians and archaeologists commonly regard the fourth-century Goths north of the lower Danube as politically and archaeologically rooted in this world, and a Wielbark contribution to Chernyakhov remains a major model (Heather, 1996; Kokowski, 2007; Andrzejowski et al., 2019; Bierbrauer, 1994). However, Chernyakhov is also reconstructed as a polyethnic formation involving Germanic-associated groups, Sarmatian and Alan steppe elements, and Dacian/Getic and other local populations (Kulikowski, 2007; Halsall, 2007; Oltean, 2007). Recent genome-wide data from the North Pontic region agree with this: Chernyakhov-associated individuals are genetically heterogeneous, including more northern/central European profiles and southern European profiles (Järve et al., 2019; Saag et al., 2025). Therefore, “from Chernyakhov” can be used mostly as shorthand for a formation zone and not for descent from a homogeneous Gothic population.

For the Balkans, the problem becomes still sharper. The lower Danube was a Roman frontier zone shaped by raiding, captivity, military recruitment, foederati settlements, ecclesiastical organisations, intermarriage, and post-Hunnic political restructuring (Heather, 1991; Heather, 1996; Wolfram et al., 1988). Gothic-associated groups entered this world through multiple historically documented episodes: third-century raids and movements across Moesia and Thrace (Jordanes, 1915; Zosimus, 1982), the Tervingian settlement associated with Ulfilas (Heather, 1986; Thompson, 2008; Heather and Matthews, 1991), the mass Danube crossings of 376 CE (Ammianus Marcellinus, 1935; Heather, 1991), the 382 CE foederati settlement, and the post-Nedao reorganisation of the later fifth century (Wolfram et al., 1988; Heather, 1991). All these events did not necessarily carry the same demographic profile. The appropriate question is, therefore, not whether the Balkan Goths had a single genome but which ancestry streams, admixture dates, and kinship patterns are associated with specific Gothic-context cemeteries.

### 1.2 Three models tested in this paper

The three historical models and their genomic expectations are summarized in Table 1. We frame the analyses around three historically meaningful but non-exclusive models. The first is a migrationist continuity model, which has been defended in recent migrationist scholarship (Heather, 1996; Kokowski, 2007; Andrzejowski et al., 2019; Bierbrauer, 1994). It predicts that at least some Balkan Gothic-associated burials should preserve a meaningful northern/Pontic component, traceable through Wielbark and Chernyakhov-related proxies, and that sites belonging to the same Gothic horizon may share broadly similar ancestry profiles.

**TABLE 1:** Formation models and genomic expectations tested in this study.

Model	Historical expectation	Genomic expectation	How the present study tests it
Migrationist continuity	Substantial movement from northern/Pontic Gothic horizons into the Balkans	Strong Wielbark/Chernyakhov-related component; possible similarity between sites	PCA, f3, qpAdm source attribution, and DATES north-source specificity
Wenskus–Wolfram–Pohl tradition-bearing model	Gothic identity maintained by tradition-bearing groups and roman political/religious contexts across changing substrates	Shared cultural label can coexist with site-specific ancestry; some continuity without genetic uniformity	Comparison of ancestry, kinship, and site structure under shared material/ritual attribution
Frontier formation (Kulikowski)	Gothic affiliation produced or expanded in roman frontier conditions	High heterogeneity, local Balkan/Roman-provincial ancestry, weak single-source continuity	Controls against local roman balkan populations; f4 and qpAdm tests of Anatolian/Balkan components

The second is a Wenskus–Wolfram–Pohl tradition-bearing model, which is named here without using the contested shorthand that is sometimes applied in historiographic literature (Wenskus, 1961; Wolfram et al., 1988; Wolfram et al., 1990; Pohl and Heydemann, 2013; Geary, 2002; Geary, 2016). Here, Gothic identity is not treated as the biological continuity of an unchanging people. Rather, names, traditions, law, political leadership, religion, language, and Roman interaction could create and maintain a gens across changing demographic substrates (Pohl and Heydemann, 2013; Heather, 1996). In Wolfram’s formulation, whole peoples do not migrate unchanged; rather, bearers of successful traditions can become founders of new ethnica (Wolfram et al., 1988; Wolfram et al., 1990). For genetics, this model predicts that Gothic-associated contexts may preserve some demographic continuity, but they need not show one stable genome-wide profile across sites or phases.

The third is a frontier-formation model that is useful as a methodological control and most clearly articulated by Kulikowski (2007). In this view, “Gothic” in the Balkans may have been expanded, stabilized, or even partly generated by military service, legal status, religious networks, and Roman categories (Kulikowski, 2007; Halsall, 2007; Goffart, 1988). Genetically, this model predicts strong site-specific heterogeneity, substantial local Balkan and Roman-provincial ancestry, and weak or absent biological continuity from a single northern source. The model does not deny the movement of people; it denies that the archaeological or textual label alone guarantees descent from a single migrating population.

These three models are not mutually exclusive but complementary expectations against which the same data can be evaluated; our framing asks which combination best explains the Bulgarian data and not which single model wins.

### 1.3 The two Bulgarian contexts

**FIGURE 1:**
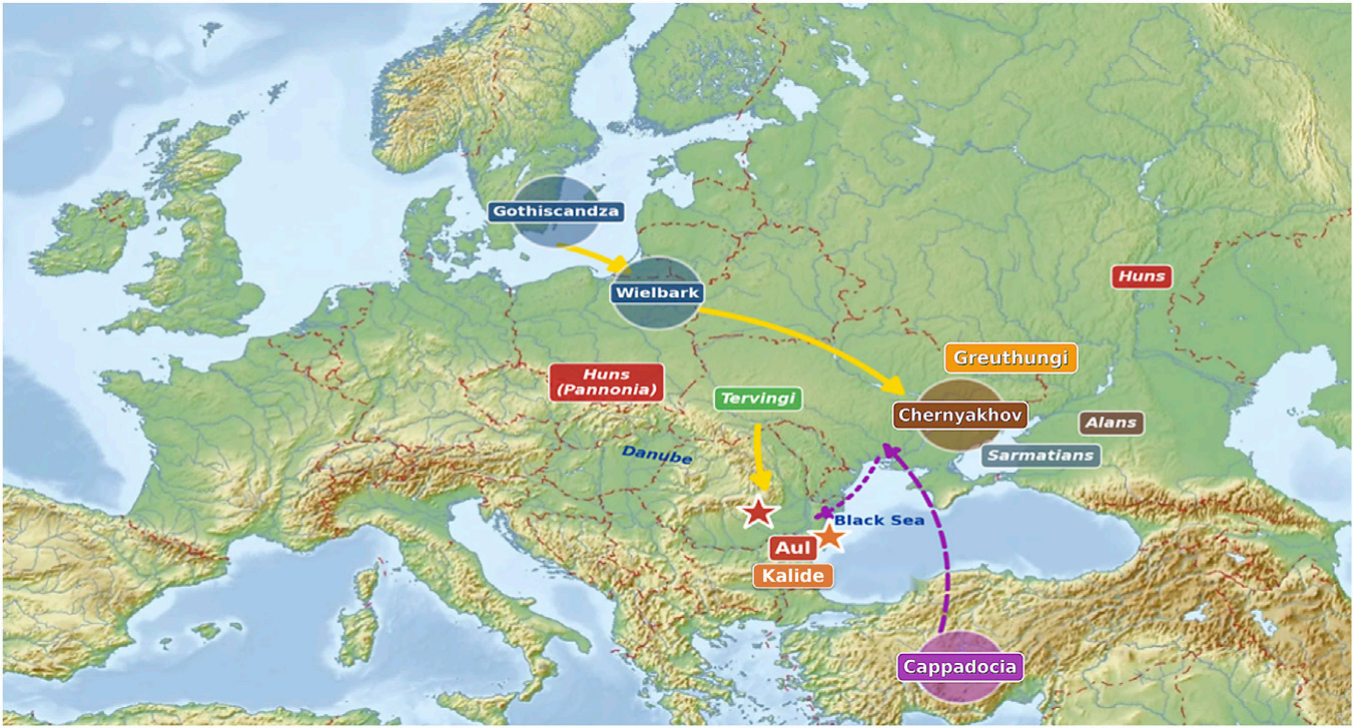
Site locations and major Gothic movement corridors referenced in the text. Aquae Calidae and the Aul of Khan Omurtag are shown within the wider lower-Danubian region and Black Sea corridor that is relevant to Gothic-period movements and contacts.

The two sites sampled here are complementary rather than interchangeable (Figure 1). Aquae Calidae, near modern Burgas on the Black Sea coast, is a fourth-century necropolis adjacent to the Roman thermal complex of Anchialos. The burials cut through or ignore the earlier Roman bath architecture, indicating reuse of a monumental Roman setting as a Christian cemetery. The assemblage includes east–west Christian burials and artefacts commonly discussed within a Gothic or wider East Germanic horizon, including costume-related and grave-good comparanda (Bierbrauer, 1994; for the full archaeological description, chronology and site plan, see Supplementary Note S3 and Supplementary Figure S3.2). The historical tradition places Gothic movements in the broader Anchialos–Aquae Calidae region in the later third century (Jordanes, 1915; Wolfram et al., 1988), but that reference concerns the region rather than the excavated cemetery itself. Aquae Calidae is, therefore, analysed here as a late Roman Christian burial community embedded in a Gothic-associated archaeological and historical horizon, while the ancestry and kinship structure of the individuals buried there are treated as empirical questions.

The Aul of Khan Omurtag (AKO), near Shumen in Moesia Secunda, is best known for its ninth-century Bulgarian monumental phase, but the Late Antique Christian complex beneath and around it has long been connected in Bulgarian scholarship with the Gothic Christian landscape of Moesia and with the community associated with Ulfilas (Balabanov, 2006; Balabanov, 2010; Heather and Matthews, 1991; Thompson, 2008). The site includes basilicas and sequential burial areas, with phases spanning the mid-fourth to later fifth century (Balabanov, 2006; Balabanov, 2010; Bierbrauer, 1994; for the per-church chronology and the burial-area sequence, see Supplementary Note S3 and Supplementary Figures S3.3, S3.4). At the same time, the site should not be fitted into a single horizon. The Late Antique church-and-cemetery complex is analytically distinct from the later medieval Bulgarian aul, and the strongest published evidence for Gothic attribution rests on a limited but important group of East Germanic or Gothic-associated funerary indicators, especially fibulae and associated dress assemblages in church-related contexts. Later burial areas require particular caution because the site was reused and some C5-context burials may belong to a later medieval horizon (Wolfram et al., 1988; Heather, 1991). This makes AKO valuable for studying continuity, transformation, and reuse, but it also requires conservative sample selection.

The two sites, therefore, test whether a shared Gothic-associated cultural and ritual horizon corresponds to shared genome-wide ancestry. Aquae Calidae represents a coastal Thracian context linked to a Roman bath complex and to a region of historically attested Gothic movement (Jordanes, 1915). AKO represents a longer-lived inland Christian centre in Moesia Secunda, within the lower-Danubian world of Ulfilas, the Gothi minores, federate politics, and post-Hunnic reorganisation (Heather, 1991; Heather and Matthews, 1991; Thompson, 2008; Wolfram et al., 1988). If the same population-history process underlay both communities, we would expect broad genomic continuity across the sites. If Gothic affiliation incorporated different local and incoming groups, the data should show site structure, sub-clusters, and possibly different admixture histories despite similar archaeological framing.

### 1.4 Aims of the present study

We report genome-wide data from a conservatively filtered set of 37 individuals from Aquae Calidae (n = 20) and AKO (n = 17) (Supplementary Tables S1, S2). The study asks four questions. First, do individuals from the two Gothic-associated Bulgarian contexts form a single genetic population, or do they differ by site and sub-cluster? Second, which ancestry components best explain the observed structure when PCA, f_3_/f_4_ statistics, and qpAdm are evaluated together? Third, when did admixture between northern/Pontic and southern Balkan–Anatolian-related components occur, and is that timing compatible with documented phases of Gothic–Roman contact, federate settlement, or earlier frontier interaction? Fourth, do close biological relationships connect the two sites, or are family relationships local to each cemetery?

We, therefore, do not ask whether these individuals were genetically “Gothic”. The archaeological record defines the contexts as Gothic-associated; the genomic record tests the demographic content of that association. Our aim in this study is to reconstruct the ancestry structure, admixture chronology, and kinship organisation in two communities situated within the Gothic-associated history of late Roman Bulgaria and to evaluate which combination of formation models best explains those demographic constraints.

## 2 Results

### 2.1 Dataset overview and conservative sample definition

The present analyses include 37 individuals, 20 from Aquae Calidae and 17 from AKO. This final dataset is smaller than the originally screened series of 53 samples. The conservative sample-selection process is summarized in Table 2. The reduction was deliberate. During archaeological and genomic review, 16 of the 53 screened individuals were set aside (per-individual record in Supplementary Table S2). Thirteen were from the C5 burial area at AKO and were judged to belong to a different, later horizon: archaeological reassessment identified this area as a probable Pecheneg-period reuse, with several of these individuals being directly dated to 700 CE–900 CE, and READv2 linked some of them to that later group (pair-level kinship in Supplementary Table S3). The remaining three from Aquae Calidae were museum-held samples accessioned as ‘Calide’ but without verifiable excavation context, and they were not used. At the end of this review, all C5 individuals were excluded except three, for which archaeological context and genomic behaviour supported retention within the Gothic-associated late antique horizon. The detection of an apparent later-horizon component within the C5 burial area is informative: archaeogenomic kinship analysis can identify cross-horizon mixing that archaeological assessment alone had not separated, and it sets the stage for a separate study of the excluded individuals. The final 37-individual set is, therefore, intended to be conservative rather than maximal.

**TABLE 2:** Conservative sample selection from the initially screened 53 individuals to the final 37-individual Gothic-associated dataset.

Stage	Decision	Rationale	Effect on the dataset
Initial screened series	A total of 53 individuals from Aquae Calidae and AKO	All available samples initially considered from the two sites	Starting dataset
First exclusion pass	Thirteen C5-area AKO individuals excluded as being part of a later horizon	Probable Pecheneg-period reuse of burial area C5 (archaeological reassessment, K. Stoeva); several individuals directly dated to 700 CE–900 CE; READv2 linked some to the later group	Removed from the Gothic-period analyses; reserved for a separate study
Second exclusion pass	Three Aquae Calidae BS-context individuals excluded	Museum-held samples accessioned as ‘calide’ but with no verifiable excavation context (no necropolis, grave, or stratigraphy)	Removed from the analytical series
Final C5 restriction	Only three C5 individuals retained	Final dataset limited to individuals with the strongest archaeological and genomic grounds for late antique Gothic-horizon assignment	Final analysed series: 37 individuals (Aquae Calidae n = 20; AKO n = 17)

All retained individuals met the analytical inclusion threshold of at least 30,000 covered autosomal SNPs after duplicate removal and contamination screening. The median 1,240 K coverage was approximately 0.6x (range c. 0.2x–2.6x; per-sample coverage in Supplementary Table S1). Genetic sex was determined for all 37 individuals (25 male and 12 female). Y-chromosome terminal calls at diagnostic positions were obtained for all 25 males (per-individual Y- and mtDNA haplogroup calls in Supplementary Tables S1, S2). The canonical sample list, coverage metrics, mtDNA and Y-DNA haplogroups, first-degree relative flags, and inclusion/exclusion status for population-level analyses are provided in Supplementary Tables S1, S2.

Two retained individuals required special handling. I41148 at Aquae Calidae had a contaminated library, so the replicate library I41148_d was used for PCA projection. I40356 at AKO had low coverage and is a first-degree relative of I41199 (see Supplementary Tables S2, S3). Both are shown in appropriate individual-level analyses but excluded from population-level qpAdm and DATES, where over-representation or low coverage could bias the pooled estimates.

### 2.2 The two sites form genetically distinct clusters

**FIGURE 2:**
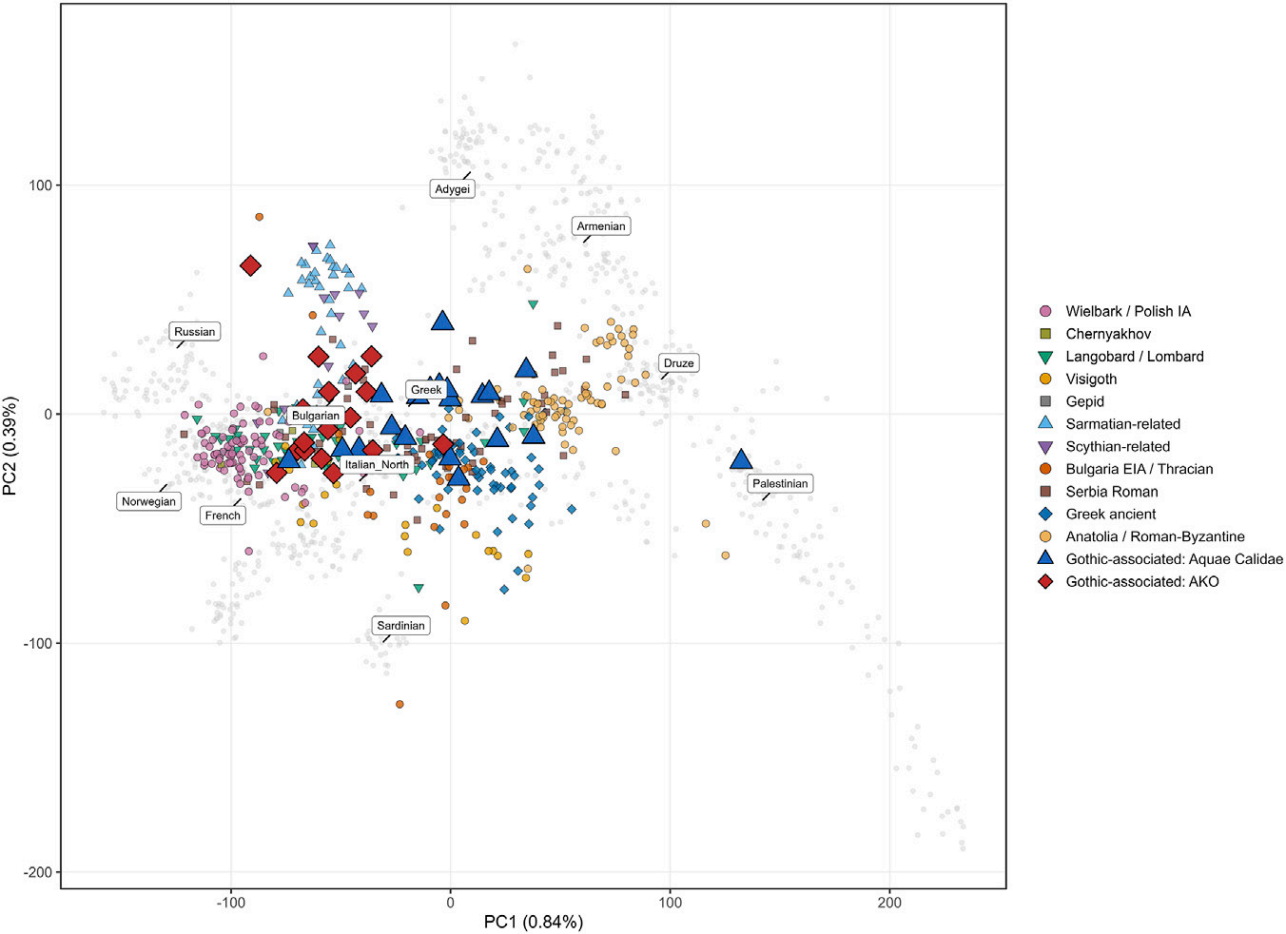
PCA of the 37 retained Gothic-associated individuals projected onto modern West Eurasian variation. Components were computed from 874 HumanOrigins modern West Eurasian individuals from 60 populations; ancient individuals and comparator groups were projected. PC1 and PC2 explain 0.84% and 0.39% of full-trace variance. Grey points show modern references; coloured points show ancient comparators; blue and red symbols mark Aquae Calidae and AKO, respectively. Modern reference populations are listed in Supplementary Table S4, ancient comparator macro-groups are listed in Supplementary Table S5, and individual-level PC coordinates and retained-sample annotations are listed in Supplementary Table S2. See also Supplementary Figure S2A.

Projected PCA onto modern West Eurasian variation (modern reference panel in Supplementary Table S4; ancient comparator macro-groups in Supplementary Table S5) reveals pronounced differences in structure between the two sites (Figure 2). In the final rerun used here, PC1 and PC2 explain 0.84% and 0.39% of the full-trace variance, respectively, with the grey background representing 874 HumanOrigins modern West Eurasian individuals from 60 populations; selected modern population centroids are labelled in Figure 2, with a fuller set of modern-population anchors for spatial orientation shown in Supplementary Figure S2A. Aquae Calidae is more dispersed than AKO and separates into two within-site sub-clusters used in the downstream analyses—a northern group (PCA North, n = 8) shifted towards central/northern European and Pontic-related variation and a southern group (PCA South, n = 11) shifted towards Balkan-, Aegean-, and Anatolian-related variation—together with a single strongly displaced individual, I40570, which falls far towards Levantine-related variation and is analysed separately (sub-cluster and outlier assignments in Supplementary Table S2). AKO forms a comparatively compact cluster aligned with Iron Age Wielbark, Polish Iron Age, and Chernyakhov-related reference variation. One AKO individual, I41202 (female; no Y-haplogroup call), is displaced from the main AKO distribution toward Sarmatian- and Scythian-related reference variation and is treated as a within-site PCA outlier (sub-cluster AKO_oNorth in Supplementary Table S2). A second AKO male, I41205, projects within the main AKO cluster on PC1/PC2 but carries the steppe/East Asian Y-haplogroup C-Y11606 (C2a1a1b1b1) (see Supplementary Tables S1, S2).

Each site contains internal variation, but the PCA separates Aquae Calidae and AKO into distinct positions despite their shared material and ritual features.

### 2.3 Outgroup f_3_ statistics support site-specific affinities

Outgroup f_3_ statistics of the form f_3_(Old_Africa1; Target, Reference) provide a descriptive measure of shared genetic drift between each Gothic-associated target and a set of ancient reference populations. Full statistical outputs are provided in Supplementary Table S6, and comparator-class interpretation is discussed in Supplementary Note S5. These statistics complement the PCA by quantifying the broad affinity structure, but they do not, by themselves, identify direct source populations or migration routes.

Aquae Calidae shows its strongest average affinities to Bulgaria Late Antiquity and related Balkan/Roman-period references, consistent with a stronger southern Balkan, Roman-provincial, or Anatolian-related component. AKO ranks these local Late Antique Balkan references lower and instead shows relatively stronger affinity to Wielbark/Polish Iron Age, Chernyakhov, and other northern/Pontic or Migration-period comparators. Both sites show low affinity to East Asian-shifted steppe comparators at the cemetery level, indicating that such ancestry is not a dominant component of the retained assemblage as a whole, although individual-level steppe/East Asian-related signals are present in specific AKO individuals.

**FIGURE 3:**
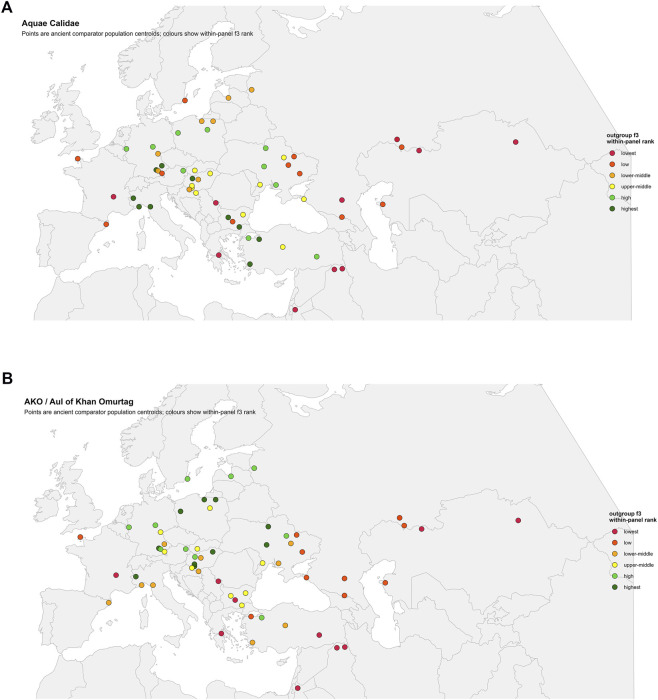
**(A,B)** Geographic visualization of outgroup f_3_ affinities for Aquae Calidae and AKO. Points represent ancient comparator populations placed at population-level coordinate centroids. Colours indicate within-panel rank of outgroup f_3_ values of the form f_3_(Old_Africa1; Target, Reference), with greener tones representing higher shared drift and redder tones representing lower shared drift relative to other mapped comparators in the same panel. **(A)** Aquae Calidae. **(B)** AKO. The map is intended as a visualization of the affinity structure and should not be interpreted as a migration route or direct source identification. Full population labels, f_3_ estimates, standard errors, Z-scores, and ranks are provided in Supplementary Table S6.

The geographic distribution of the f_3_ affinities is summarized in Figure 3. The map plots ancient comparator populations at population-level coordinate centroids where coordinates were available and colours them by the within-panel f_3_ rank. This visualization is intended to make the site-level affinity contrast legible rather than to imply geographic origins or migration routes; exact f_3_ estimates, standard errors, Z-scores, ranks, and population labels are provided in Supplementary Table S6.

Aquae Calidae is shifted more strongly towards southern Balkan/Anatolian-related and Roman-provincial comparators, whereas AKO shows a stronger northern/Pontic-affinity profile. The numerical differences in f_3_ are modest and should be interpreted as relative affinity shifts rather than direct source identifications.

### 2.4 qpAdm confirms site-specific source structures at Aquae Calidae and AKO

qpAdm was run on analysis-specific population-level target definitions anchored to the final retained sample set. The population-level targets are Aquae Calidae PCA South (n = 11), Aquae Calidae PCA North (n = 8), the Aquae Calidae outlier I40570 analysed separately, AKO pooled excluding the low-coverage individual I40356 (n = 16), AKO C4 (n = 11), and AKO C5 (n = 3). The complete proximal qpAdm model set, including full requested models, best feasible nested/popdrop models, weak contrasts, and rejected one-source controls, is reported in Supplementary Table S7. Individual-level qpAdm results filtered to the final retained sample set are reported in Supplementary Table S8, and distal qpAdm checks are reported in Supplementary Table S9 and Supplementary Figure S9.

The two Gothic-associated assemblages are not described by the same source structure. Aquae Calidae cannot be reduced to a single Wielbark/Polish Iron Age or Chernyakhov-related source. Its PCA South sub-cluster accepts models combining a southern Anatolian-related proxy, Balkan Iron Age ancestry, and a Chernyakhov-related component, while models without an Anatolian-related proxy are weak and one-source Wielbark/Polish Iron Age or Chernyakhov controls are rejected. Aquae Calidae PCA North also rejects single-source northern/Pontic models: a nested Iznik/Marmara plus Chernyakhov-outlier model is feasible, and a Kalehoyuk + Bulgaria_EIA + Chernyakhov contrast model is also accepted. Thus, the data do not support a rigid Kalehoyuk-versus-Iznik split within Aquae Calidae; the supported conclusion is that both Aquae sub-clusters require southern Anatolian/Marmara-related ancestry together with a northern/Pontic-related component.

AKO is structurally different. The pooled AKO target excluding I40356 is well-modelled by Chernyakhov-related and Balkan Late Antique ancestry, and the same broad structure fits AKO C4 and C5. C4 is more strongly Chernyakhov-related, whereas C5 shows a larger local/Balkan Late Antique contribution; because C5 has only three retained individuals and the site has a complex reuse history, this difference is treated as suggestive rather than as a formal temporal trend. Sensitivity models including Early Sarmatian ancestry are tolerated, especially for C4, but they do not replace the simpler conclusion that AKO is more northern/Pontic-shifted than Aquae Calidae.

All qpAdm source labels are proxy-defined ancestry streams and not literal source communities. Kalehoyuk, Iznik/Marmara, Chernyakhov, and Wielbark/Polish Iron Age are used to distinguish southern Anatolian/Marmara-related, northern/Pontic-related, and local Balkan components within the available reference panel. The evidential weight lies in the contrast between accepted multi-source models and weak or rejected alternatives, not in any single proxy name.

In the selected full requested and best feasible nested/popdrop models, “Full” denotes the requested source combination and “nested/popdrop” denotes the best feasible subset recovered when one source was not required or produced an infeasible coefficient. Proportions are rounded to one decimal percentage. Complete results are reported in Supplementary Table S7.

Together, these models show that qpAdm supports the same site-level contrast observed in PCA and outgroup f_3_ and f_4_ statistics. AKO and parts of Aquae Calidae preserve substantial northern/Pontic-related ancestry, Aquae Calidae also requires a southern Anatolian/Marmara-related component, and the two sites are not described by the same source structure. The historical interpretation of these contrasts is developed in the Discussion.

#### 2.4.1 Individual-level qpAdm and within-site heterogeneity

Individual-level qpAdm models are retained as descriptive evidence for within-site heterogeneity but are not used as the primary basis for source attribution. Single-individual qpAdm is sensitive to coverage, source choice, and overfitting; therefore, the main text uses group-level qpAdm as the evidential base and treats individual-level models as supporting documentation in Supplementary Table S8.

The individual-level results are most useful for illustrating diversity within the same archaeological horizon. I40570 remains a strong outlier: it is not adequately modelled by the standard Aquae Calidae/AKO source set and should not be allowed to drive site-level Aquae Calidae models. Within AKO, individual and small-sub-cluster models document additional C4 heterogeneity, including steppe/East Asian-shifted individuals, but these are best treated as sensitivity evidence rather than as the main population-level result. Paternal lineages may track part of the autosomal heterogeneity at Aquae Calidae, but the retained sample definition and the population-level qpAdm behaviour do not support lineage-stratified qpAdm as a central claim.

#### 2.4.2 Distal qpAdm as a descriptive deep-ancestry check

Distal qpAdm was used as an independent deep-ancestry check rather than as a primary source-attribution framework. The proximal models discussed above directly test the historically relevant alternatives: northern/Pontic-related ancestry, local Balkan/Roman-period ancestry, and southern Anatolian/Marmara-related ancestry. Distal components such as Turkey_N, CHG, EHG, WHG, Levant_PPN, or Cayonu_PPN are, therefore, treated as summaries of the deep ancestry structure and not as literal Late Antique source populations.

The distal results confirm broad differences in the deep ancestry structure between the two sites. Aquae Calidae PCA South accepts a Turkey_N + CHG + EHG + Levant_PPN + WHG model (p = 0.290), whereas Aquae Calidae PCA North accepts a Cayonu_PPN + CHG + EHG + WHG model (p = 0.178). AKO pooled excluding I40356, AKO C4, and AKO C5 accept Turkey_N-centred models (p = 0.284, 0.298, and 0.289, respectively), with C4 tolerating a very small SlabGrave-related term in the selected distal model. I40570 has no accepted distal model under the tested panel and is, therefore, reported as an outlier audit result rather than plotted as an accepted fit.

**FIGURE 4:**
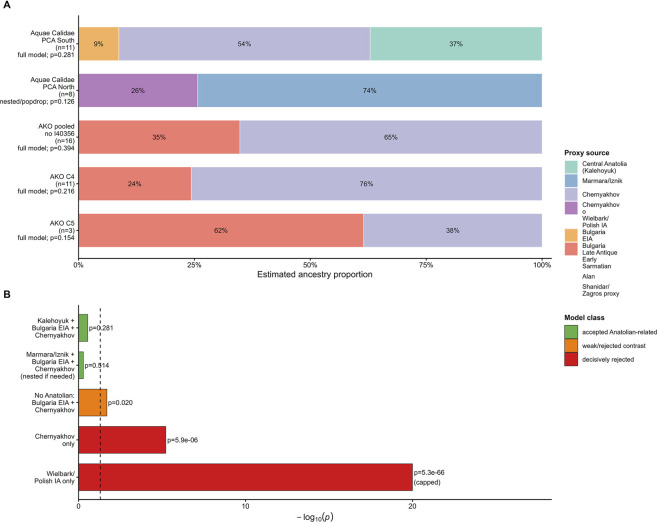
Proximal qpAdm models distinguish between Aquae Calidae and AKO. **(A)** Representative corrected proximal qpAdm models for the final retained Gothic-associated targets: Aquae Calidae PCA South (n = 11), Aquae Calidae PCA North (n = 8), AKO pooled excluding I40356 (n = 16), AKO C4 (n = 11), and AKO C5 (n = 3). Bars show the estimated ancestry proportions in selected accepted or nested feasible models. Chernyakhov is used here as the principal proximal northern/Pontic proxy in the representative accepted models; Wielbark/Polish IA is retained as a contrast model and reported in Supplementary Table S7. **(B)** Model-fit contrasts for Aquae Calidae PCA South show accepted Anatolian-related models against weak or rejected no-Anatolian, Chernyakhov-only, and Wielbark/Polish IA-only alternatives. Source labels are proxy populations and should not be interpreted as literal source communities. Full requested models, nested/popdrop results, p-values, feasibility status, and model definitions are provided in Supplementary Table S7.

The full distal model set is reported in Supplementary Table S9 and visualised in Supplementary Figure S9. Distal qpAdm here is supportive and descriptive: it shows that the two sites differ in broad deep-ancestry composition, while the historical interpretation rests on the proximal qpAdm results shown in Table 3 and Figure 4.

**TABLE 3:** Proximal qpAdm models for retained Gothic-associated groups.

Target	Model/Sources	Fit	Proportions	Interpretation
Aquae Calidae PCA South (n = 11)	Accepted full model: Kalehoyuk + Bulgaria_EIA + Chernyakhov	p = 0.281; feasible	37.1% + 8.7% + 54.2%	Southern Anatolian-related proxy + northern/Pontic component fits
Aquae Calidae PCA South (n = 11)	Alternative nested contrast: Iznik/Marmara + Bulgaria_EIA + Chernyakhov	Full p = 0.461 but infeasible; nested p = 0.514	Full model has Bulgaria_EIA ≈ −0.2%; nested model is Iznik/Marmara 49.2% + Chernyakhov 50.8%	A broader southern Anatolian/Marmara-related proxy can also capture the southern component; avoid over-claiming a unique Kalehoyuk source
Aquae Calidae PCA South (n = 11)	Weak no-Anatolian contrast: Bulgaria_EIA + Chernyakhov	p = 0.020	46.1% + 53.9%	Falls below the usual p > 0.05 threshold; supports the need for a southern Anatolian/Marmara-related proxy
Aquae Calidae PCA South (n = 11)	Rejected one-source controls: Chernyakhov only; Wielbark/Polish IA only	p = 5.94 × 10^−6^; p = 5.29 × 10^−66^	100%; 100%	Not explained by a single northern/Pontic proxy
Aquae Calidae PCA North (n = 8)	Full model is unstable; nested model is feasible: Iznik/Marmara + Chernyakhov + Chernyakhov_o	Full p = 0.095, infeasible; nested p = 0.126	Full: Iznik/Marmara 74.3%, Chernyakhov −1.4%, Chernyakhov_o 27.1%; nested: Iznik/Marmara 74.3% + Chernyakhov_o 25.7%	Marmara/Iznik-related + Chernyakhov-outlier/Pontic-related ancestry captures the sub-cluster; ordinary Chernyakhov is not required in this full model
Aquae Calidae PCA North (n = 8)	Accepted contrast: Kalehoyuk + Bulgaria_EIA + Chernyakhov	p = 0.139; feasible	58.1% + 18.4% + 23.6%	Data do not support an absolute Kalehoyuk-versus-Iznik separation
Aquae Calidae PCA North (n = 8)	Rejected one-source controls: Chernyakhov_o only; Wielbark/Polish IA only	p = 1.04 × 10^−11^; p = 2.30 × 10^−115^	100%; 100%	Not a single northern/Pontic source
Aquae Calidae outlier I40570 (n = 1)	Failed standard model: Iznik/Marmara + Bulgaria_LAntiquity + Chernyakhov	Full p = 0.004, infeasible; nested Chernyakhov-only p = 2.81 × 10^−31^	Extreme full-model weights	Not captured by the standard site-level source set; treated separately
AKO pooled, excluding I40356 (n = 16)	Accepted full model: Chernyakhov + Bulgaria_LAntiquity	p = 0.394; feasible	65.2% + 34.8%	Well-described by a Chernyakhov-related + Balkan Late Antique structure
AKO pooled, excluding I40356 (n = 16)	Sensitivity model: Chernyakhov + Bulgaria_EIA + early Sarmatian	Full p = 0.109; nested p = 0.134	Full: 63.6% + 30.0% + 6.4%; nested drops early Sarmatian	A small steppe-related term is tolerated but not required in the pooled model
AKO C4 (n = 11)	Accepted full model: Chernyakhov + Bulgaria_LAntiquity	p = 0.216; feasible	75.7% + 24.3%	C4 is more strongly Chernyakhov-related than the pooled AKO average
AKO C4 (n = 11)	Sensitivity model: Chernyakhov + Bulgaria_EIA + early Sarmatian	p = 0.110; feasible	60.4% + 25.2% + 14.4%	Steppe-related ancestry is plausible in C4 sensitivity models but should not replace the simpler two-source description
AKO C5 (n = 3)	Accepted full model; small n: Chernyakhov + Bulgaria_LAntiquity	p = 0.154; feasible	38.5% + 61.5%	Indicates stronger local/Late Antique contribution than C4, but interpretation is limited by n = 3

### 2.5 f_4_ statistics reject a Chernyakhov-only explanation for the Anatolian-related component

The qpAdm results show that Aquae Calidae requires southern Anatolian/Marmara-related ancestry in addition to northern/Pontic-related ancestry. The remaining interpretive question is whether this southern component could have arrived only as part of a Chernyakhov-derived population. f_4_ statistics argue against this interpretation.

First, Chernyakhov-associated references are shifted more strongly towards Balkan than towards Anatolian proxies in tests of the form f_4_ (Mbuti, Chernyakhov; Anatolian, Balkan). Representative positive Z-scores include Kalehoyuk versus Bulgaria_EIA (Z = +2.55), Gordion versus Bulgaria_EIA (Z = +4.33), Iznik versus Bulgaria_EIA (Z = +2.85), and Mardin versus Bulgaria_EIA (Z = +7.41) (Supplementary Table S6). If Chernyakhov is closer to Balkan references than to Anatolian references in these tests, it cannot by itself explain the stronger Anatolian-related signal observed in Aquae Calidae.

Second, Aquae Calidae South shows Anatolian-related affinity beyond the Chernyakhov baseline. In tests of the form f_4_ (Mbuti, Anatolian; Aquae_Calidae_South, Chernyakhov), multiple Anatolian proxies show significant negative values, including Gordion (Z = −3.11), Mardin (Z = −2.61), Apollonia Roman (Z = −2.30), Mugla (Z = −2.26), Iznik (Z = −2.19), and Kalehoyuk (Z = −2.09) (Supplementary Table S6). This is consistent with the qpAdm contrast in which Aquae Calidae South accepts models containing a southern Anatolian-related proxy but weakens when that proxy is removed.

Third, head-to-head f_4_ comparisons among Anatolian proxies support the use of Central Anatolian/Kalehoyuk-like sources as a useful proxy for the Aquae Calidae South signal: representative contrasts against alternative Anatolian references include Mardin (Z = −5.04), Kalehoyuk Iron Age (Z = −4.82), Van Urartian (Z = −3.77), and Gaziantep Byzantine (Z = −3.18) (Supplementary Table S6). The qpAdm results nevertheless support framing the broader conclusion as southern Anatolian/Marmara-related rather than as the literal descent from one named source. Finally, comparisons against Serbia_Roman_Rit and other Roman-period Balkan controls show that the Anatolian-related signal in Aquae Calidae is not simply equivalent to generic Roman Balkan background. Together, f_4_ statistics and qpAdm reject a Chernyakhov-only explanation while avoiding a claim of direct migration from any single Anatolian archaeological site.

### 2.6 DATES places north–south admixture before the burial horizon

**FIGURE 5:**
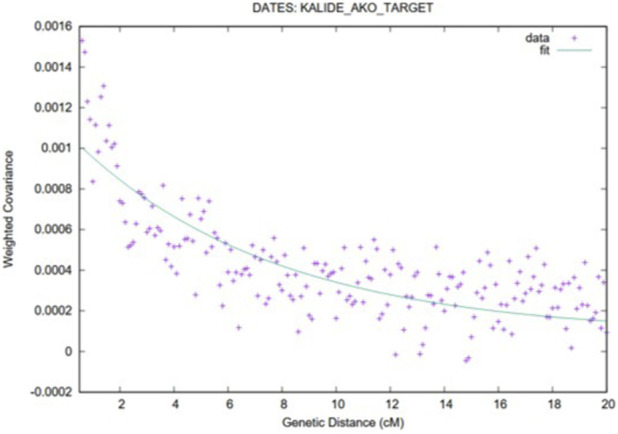
DATES admixture-dating for the main north–south signal: weighted ancestry-covariance decay curve for the headline KalideN + AKO target using a POL3/Wielbark-related northern source and a southern Balkan–Anatolian source configuration; observed decay points are fitted by an exponential curve. South-proxy rotation and pooled-source sensitivity are reported in Tables 4, 5; complete runs, parameters, and diagnostic failures are shown in Supplementary Table S10 and Supplementary Note S6.

DATES was used only after PCA, f-statistics, and qpAdm had identified plausible northern/Pontic and southern Balkan–Anatolian ancestry poles. The weighted ancestry-covariance decay for the headline KalideN + AKO run is shown in Figure 5; the south-proxy rotation and pooled-source sensitivity are reported in Tables 4, 5. The most informative pooled target, KalideN + AKO, using the POL3 Wielbark-related northern source and a southern Balkan–Anatolian source configuration, gives 12.44 ± 2.36 generations before burial (Z = 5.26; nrmsd = 0.186), corresponding to a first-century CE point estimate with a 95% confidence interval of 85 BCE–183 CE (Table 6; Supplementary Table S10; Supplementary Note S6).

**TABLE 4:** South-proxy rotation for the KalideN + AKO target with POL3 north.

South proxy	Source pool n	Mean (gen)	SE	Z	nrmsd	Calendar point
AnatoliaMax	37	12.44	2.36	5.26	0.186	c. 49 CE
Mugla3	23	14.65	3.14	4.67	0.160	c. 49 CE
BalkanCore	44	12.12	2.76	4.39	0.221	c. 49 CE

**TABLE 5:** Sensitivity of the KalideN + AKO admixture-time estimate to single versus pooled southern source configurations (POL3/Wielbark-related northern source).

Southern source configuration	Mean (gen)	SE	Z	Effect relative to single-proxy baseline
BalkanCore (single proxy)	12.12	2.76	4.39	Baseline single-proxy model
AnatoliaMax (single proxy)	12.44	2.36	5.26	Concordant with baseline (Δ < 1 generation)
BalkanCore + Mugla3 (pooled)	14.24	4.83	2.95	SE inflated ≈75%; Z reduced
BalkanCore + AnatoliaMax (pooled)	15.54	5.12	3.04	SE inflated ≈85%; Z reduced

Combining two southern proxies into a single pooled source inflates the standard error and lowers the Z-score relative to single-proxy models, indicating that the data do not resolve two separate southern admixture pulses. Single-proxy rotations are reported in Tables 4, 5; the complete runs and diagnostics are in Supplementary Table S10 and Supplementary Note S6.

**TABLE 6:** Main DATES admixture-time estimates.

Target	North	South	Mean (gen)	SE	Z	nrmsd	Calendar interpretation
KalideN + AKO	POL3 wielbark	AnatoliaMax	12.44	2.36	5.26	0.186	Point estimate c. 1st c. CE; 95% CI, 85 BCE–183 C E
KalideN	POL3	Mugla3	12.74	2.70	4.73	0.191	Comparable early Roman-period estimate
KalideN + AKO	POL3	Mugla3	14.65	3.14	4.67	0.160	Similar within uncertainty
KalideN	POL3	BalkanCore	12.09	2.61	4.63	0.318	Comparable
KalideN	POL3	AnatoliaMax	11.01	2.42	4.55	0.242	Comparable
KalideN + AKO	POL3	BalkanCore	12.12	2.76	4.39	0.221	Comparable
AKO alone	Chernyakhov	Mugla_Stratonikeia	17.23	3.94	4.38	0.454	Older and less stable; interpreted cautiously

The north-source tests show that the signal is strongest when the northern pole is anchored in Polish Iron Age/Wielbark-related sources, especially Pruszcz Gdański, Masłomęcz, and Kowalewko. Adding broader Scandinavian Iron Age samples shifts the date earlier and inflates uncertainty, indicating that the informative contrast is not simply generic northern European ancestry. The south-source rotation shows that BalkanCore, AnatoliaMax, and Mugla3 converge within the standard errors of the estimates, whereas the union-south configurations inflate the standard errors and reduce Z (Table 5; Supplementary Table S10). Because pooling two southern proxies into a single source yields no improvement in fit, a single southern source is sufficient to describe the signal; therefore, the data do not resolve two separate southern admixture pulses and are most consistent with a single north–south admixture process shortly before the burial horizon (Table 5).

#### 2.6.1 Specificity controls: the signal is absent in non-Gothic Roman Balkan populations

The DATES signal is specific to the Gothic-associated target configuration. Serbia_Viminacium_Roman_Rit (n = 3) and additional Roman-period Bulgarian populations failed all DATES contrasts under the same parameters, with Z < 1.8 and nrmsd >0.66 (Supplementary Note S6, §S6.7). The ∼12-generation signal with Z = 4.4–5.3 is not recovered in any non-Gothic Roman-period Balkan population tested with comparable source rotations. This is directly relevant to the model frame: if the signal were a generic regional artefact of the Roman–Balkan population structure, it should also appear when these control populations are placed as targets against the same north and south reference poles. It does not. Therefore, the DATES signal reflects the Gothic-associated north–south ancestry contrast specifically and not the broader Anatolian-shifted Roman Balkan background documented by Olalde et al. (2023). Combined with the f_4_ evidence in §2.5, this excludes a single Chernyakhov-carrier explanation for the Anatolian-related component and constrains the frontier-formation model: the formation processes operating at Aquae Calidae and AKO produced a quantitatively different ancestry pattern from the comparable non-Gothic Roman Balkan controls.

### 2.7 Uniparental markers corroborate heterogeneity

Y-chromosome and mitochondrial haplogroups add independent support for ancestry heterogeneity, although they are not interpreted as population ancestry by themselves. Among males with terminal Y-haplogroup calls, northern/central European-associated lineages (I1, R-DF90, and I-Y22019) occur alongside Anatolian/Near Eastern lineages (J1 and J2a clades, G-FT108576), the Balto-Slavic R1a-M458/L1029 lineage R-YP593, and two steppe/East Asian C2a lineages (C-F9721 in I41149 and C-Y11606 in I41205) (per-individual Y- and mtDNA calls in Supplementary Tables S1, S2). The distribution is not consistent with a single patrilineal founder group. At AKO, Y-haplogroup composition shifts across phases: earlier phases are richer in northern European lineages, while two of two retained C5 males with determined Y-haplogroups carry Anatolian/Near Eastern lineages (95% CI 16%–100%; sample size precludes population-level inference). mtDNA haplogroups are similarly diverse and include lineages compatible with local Balkan continuity (H, J, T2, K, HV, V, X2, W, I) as well as broader Mediterranean and steppe-related mobility (U5, U4a2, and U2e1f1) and rarer Near Eastern (U7b, N1a1a3, and N1b1a), East Eurasian (C5a1), and African (L1b1a) signals. Because coverage varies by individual, terminal sub-clade interpretation is restricted to calls supported by diagnostic SNP coverage. Uniparental markers do not contradict the autosomal evidence for biological heterogeneity within the Gothic-associated burial horizon, and the Y-versus-mtDNA contrast at AKO is consistent with, although not proof of, male-biased demographic processes (Veeramah et al., 2018).

### 2.8 Kinship is local and not inter-site

**FIGURE 6:**
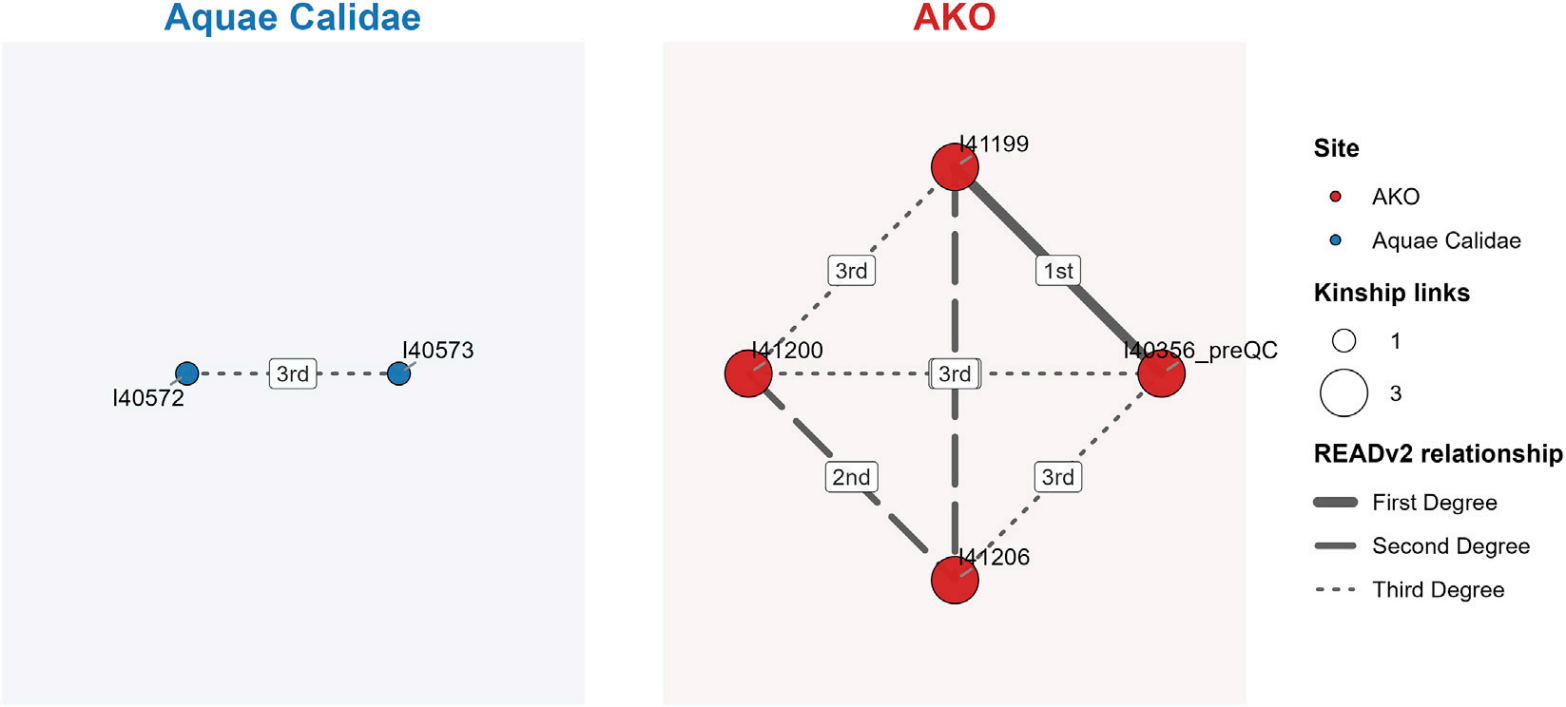
READv2 kinship network among retained and excluded individuals. Nodes represent sampled individuals, with retention status and distinguished site assignment; edges mark first- and second-degree READv2 relationships. Complete pair-level calls and sensitivity summaries are provided in Supplementary Table S3.

READv2 kinship analysis (Monroy Kuhn et al., 2018) identifies close biological relationships within sites but no confirmed close kinship connecting Aquae Calidae and AKO [Figure 6; complete pair-level output is shown in Supplementary Table S3 (sheet S3_All_1st_to_3rd, with within-site, comparator, and overlap subsets flagged in its Subset_membership column, and cross-site pair status in sheet S3_Candidate_Status); analytical discussion is provided in Supplementary Note S4). This is significant because the two sites share archaeological framing but not close family networks in the sampled data. Therefore, kinship supports the interpretation that these were distinct local communities participating in overlapping Gothic-associated cultural and religious horizons rather than one extended family network moving between cemeteries. In the filtered dataset, close-relative handling was also used to prevent over-representation of single families in population-level qpAdm, f-statistics, and DATES.

The kinship results also contributed to sample filtering. READv2 identified close relationships involving individuals suspected to belong to the later-horizon group in the C5 burial area (see Supplementary Table S3, sheet S3_All_1st_to_3rd, where within-Gothic pairs are flagged in the Subset_membership column). This did not by itself determine the archaeological dating, but it strengthened the decision to exclude uncertain C5 individuals and retain only three individuals with the strongest evidence for assignment to the late antique Gothic-associated horizon (final retention rationale is provided in Supplementary Table S2). Therefore, kinship was used both analytically and as a quality-control tool for horizon definition—a methodological point that may itself be useful for future archaeogenomic work on multi-phase cemetery complexes.

## 3 Discussion

### 3.1 The data reject a single biological Gothic profile

The most direct conclusion is negative but important: the two Gothic-associated Bulgarian assemblages do not represent a single genome-wide ancestry profile. Aquae Calidae and AKO share archaeological and ritualistic features, but PCA separates them, f_3_ ranks their closest affinities differently, qpAdm requires different source structures, and there are no close kinship links the two sites. This rejects a simple model in which a biologically coherent Gothic population moved intact into the Balkans and produced both cemeteries with only minor local admixture.

This conclusion should not be misread as anti-migration. AKO preserves a strong northern/Pontic signal, and several models require Wielbark/Chernyakhov-related ancestry. Therefore, the data also reject a strictly local model in which Gothic material culture in Bulgaria was adopted by an unchanged Roman Balkan population. The better interpretation is mixed: real northern/Pontic ancestry entered the Balkans within Gothic-associated networks, but the communities buried at Aquae Calidae and AKO were already different mixtures of northern, Pontic, Balkan, Anatolian, Near Eastern, and steppe-related ancestry.

The principal analytical methods and outputs are summarized in Table 7.

**TABLE 7:** Summary of the principal analytical methods and outputs.

Method	Software/Version	Key settings	Primary output
PCA	Smartsnp v1.2.0	Drift scaling; RSpectra SVD; ancient projection; full-trace variance denominator	Figure 2; PCA coordinates
f_3_/f_4_	ADMIXTOOLS2	Old_Africa1 outgroup for f3; block-jackknife SE; │Z│ ≥ 3 treated as strong	Figure 3; Table 8; Supplementary Table S6
qpAdm	ADMIXTOOLS2	11-Population right set (mbuti, Kostenki14, villabruna, Yana_UP, MA1, DevilsCave, karitiana, natufian, Boncuklu_PPN, GanjDareh_N, iberomaurusian); p > 0.05 feasible; p ≪ 10^−10^ decisive rejection	Figure 4; Tables 3, 8; Supplementary Tables S7–S9; Supplementary Figure S9
DATES	v4010	Binsize 0.001; mindis 0.02; maxdis 0.5; lovalfit 0.6; generation time 29 years; specificity tested against non-Gothic roman balkan controls	Tables 4–6; Figure 5; Supplementary Table S10; Supplementary Note S6
Kinship	READv2	Kinship cutoff 0.04; pair overlap ≥15,000 SNPs; first-degree handling	Figure 6; Supplementary Table S3; Supplementary Note S4
Y-DNA	yHaplo	Diagnostic SNP calls at ≥ 3× coverage; ISOGG v15.73	Supplementary Table S1
mtDNA	HaploGrep2	PhyloTree build 17	Supplementary Table S1

**TABLE 8:** Summary of f_4_/qpAdm logic rejecting a Chernyakhov-only explanation.

Test/Comparison	Result	Interpretation
f4 (Mbuti, Chernyakhov; Anatolian, Balkan)	Chernyakhov is closer to Balkan references than to Anatolian references across multiple proxy pairings (Supplementary Table S6)	Anatolian-related ancestry at Aquae Calidae cannot be explained solely as a Chernyakhov-carried signal
f4 (Mbuti, Anatolian; Aquae Calidae South, Chernyakhov)	Aquae Calidae South is shifted towards Anatolian-related proxies relative to Chernyakhov	Aquae Calidae South carries Anatolian-related affinity beyond the Chernyakhov baseline
qpAdm: Aquae Calidae south with Anatolian-related proxy	Kalehoyuk + Bulgaria_EIA + Chernyakhov fits the n = 11 target (p = 0.281)	A southern Anatolian-related proxy improves the source structure
qpAdm: Aquae Calidae South without Anatolian-related proxy	Bulgaria_EIA + Chernyakhov is weak (p = 0.020); Chernyakhov-only and Wielbark/Polish IA-only models are rejected	A northern/Pontic-only or Balkan + Chernyakhov-only model is insufficient
Aquae Calidae versus Roman-period Balkan controls	f_4_ comparisons against Roman-period Balkan controls show that the signal is not simply generic provincial Balkan background (Supplementary Table S6)	The southern component is part of the Gothic-associated target structure and not only a property of the comparison panel

### 3.2 Which formation model fits best?

The migrationist continuity model is supported only in the qualified form. It correctly predicts a northern/Pontic component, especially at AKO and in parts of Aquae Calidae. It is also compatible with the DATES signal being the strongest when the north source is anchored in Polish Iron Age/Wielbark-related groups rather than generic northern European group. However, it is not a complete explanation because Aquae Calidae is too southern and internally structured and because no single ancestry model fits all 37 retained individuals.

The Wenskus–Wolfram–Pohl tradition-bearing cultural–political model fits the overall pattern better than a single-source migration model (Wenskus, 1961; Wolfram et al., 1988; Pohl and Heydemann, 2013). The data show that Gothic-associated material and ritual practice could be shared by communities with different ancestry histories. This is the kind of demographic situation in which identity may be maintained not by biological uniformity but by traditions, legal and political affiliation, religious organisation, language, and Roman frontier institutions (Wolfram et al., 1988; Pohl and Heydemann, 2013; Heather, 1996; for the full historiographic framing of Wenskus, Wolfram, Pohl, Heather, and Kulikowski, see Supplementary Note S2). The corrected qpAdm analysis strengthens this interpretation at the population level: Aquae Calidae and AKO require different source structures, while individual-level qpAdm in Supplementary Table S8 documents additional within-site heterogeneity. Genomics cannot prove the institutional mechanisms by which the Gothic identity was maintained, but it can show that the demographic substrate was heterogeneous enough to require cultural–political mechanisms of incorporation.

The frontier-formation model in the Kulikowski tradition is also strongly relevant, especially for Aquae Calidae (Kulikowski, 2007). The southern Balkan/Anatolian-related ancestry there cannot be reduced to Chernyakhov alone; it points to Roman provincial and frontier populations already carrying mixed Balkan–Anatolian ancestry (Olalde et al., 2023; Oltean, 2007; archaeogenetic literature placement of Aquae Calidae relative to Stolarek et al., 2019; Stolarek et al., 2023; Saag et al., 2025; and Golubiński et al.,2026 in Supplementary Note S1). However, a frontier-only model would understate the northern/Pontic ancestry at AKO and parts of Aquae Calidae, and it would also fail to predict the §2.6.1 specificity controls: if the Gothic-associated signal was a generic frontier artefact, the same DATES contrast should also appear in non-Gothic Roman Balkan controls, but it does not. Therefore, the best-fitting historical reading is not one model in isolation. It includes both migration and coalition plus frontier incorporation: Gothic-associated groups reaching Bulgaria carried real northern/Pontic ancestry, but they entered a lower-Danubian world already shaped by Roman mobility, local Balkan populations, Anatolian-related ancestry, steppe elements, and previous admixture.

### 3.3 The Anatolian-related component and the pre-blended Balkan–Anatolian substrate

The Anatolian-related component is the main interpretive challenge. Historical narratives of Gothic–Anatolian contact include third-century raids and captivity, the ancestry of Ulfilas, and later Gothic movements into Byzantine Anatolia (Wolfram et al., 1988; Heather, 1991; Thompson, 2008; Procopius, 1940; Constantine et al., 1967). These are important comparanda, but the genetic data do not require a single direct Cappadocian-captive explanation for Aquae Calidae. The stronger genetic claim is more cautious: Aquae Calidae carries ancestry that available qpAdm models capture with Anatolian-related proxies, and the f_4_ statistics in §2.5 show that this component cannot be explained by Chernyakhov alone. Distal qpAdm (§2.4.2) further separates the two Aquae Calidae sub-clusters from each other: PCA North accepts a Çayönü_PPN + CHG + EHG + WHG model (p = 0.178), while PCA South does not—PCA South is accepted under a Turkey_N + CHG + EHG + Levant_PPN + WHG model (p = 0.290). The North sub-cluster, therefore, carries a Southeast Anatolian deep-ancestry signal that PCA South and the remaining targets do not (Lazaridis et al., 2016; Lazaridis et al., 2022; see Supplementary Table S9 for the full distal qpAdm matrix).

DATES then constrains the timing of the relevant north–south mixture to approximately 12 generations before burial, with a first-century CE point estimate and a 95% confidence interval of 85 BCE–183 CE. The point estimate precedes the historically documented Gothic settlement south of the Danube by approximately 100–200 years; the upper bound of the 95% confidence interval overlaps the period of the earliest documented Gothic–Roman contacts. Three readings of this temporal offset are compatible with the data, and their combination is the most parsimonious account.

First, admixture may have occurred outside the Bulgarian settlement zone—along the trans-Danubian frontier corridor (Roman Dacia, the Pontic Bosporan zone, or Chernyakhov-culture territory east of the Dniester) before the documented Gothic arrival in the lower Balkans. The Wielbark–Pontic/Aegean contact zones of the first–second centuries CE are historically and archaeologically attested, and Roman Dacia after 106 CE received documented Anatolian and central Balkan colonists (Oltean, 2007), generating exactly the kind of Balkan–Anatolian blended substrate that the qpAdm and f_4_ evidence jointly require (Olalde et al., 2023).

Second, the generation-time assumption may be too long. Late Iron Age and Roman-period populations under high adult mortality may have had effective generation times of 25–27 years rather than 29 (the project convention following Moorjani et al., 2016; sensitivity of the calendar date to this assumption is discussed in Supplementary Note S6). A 25-year generation-time shifts the headline point estimate forward by approximately 50 years (∼49 CE → ∼99 CE), and a 22–25-year average shifts it into the second century CE, approaching the documented contact horizon. The qualitative significance class is unaffected by this rescaling.

Third, the Wielbark reference may itself produce a dating effect. The POL3 sites (Pruszcz Gdański, Masłomęcz, and Kowalewko) date to the first–second century CE (Stolarek et al., 2023; placement of the Wielbark horizon in the wider archaeogenetic literature in Supplementary Note S1). If the actual Gothic migrants to the Balkans were descendants of this Wielbark-related population several generations later, the DATES “clock” references the Wielbark sampling horizon rather than the migration itself; the inferred date then represents the time that the ancestors of the migrants admixed, not when the migrants themselves arrived in Bulgaria.

These three readings are not mutually exclusive; the data are consistent with a combination of all three. Within the model frame of §1.2, each reading also tilts the interpretation slightly differently: the trans-Danubian-frontier reading shifts weight toward the frontier-formation model; the generation-time reading is approximately model-neutral; the Wielbark-reference reading is more compatible with the migrationist continuity model than the surface point estimate alone would suggest. We acknowledge this interpretive choice openly rather than collapsing it to a single explanation. Direct ancient DNA from candidate admixture locations—Roman Dacia, the Pontic-Caspian frontier of the Wielbark expansion, and Roman-period central Anatolia—will be required to distinguish among these readings.

### 3.4 AKO, C5, and the horizon definition

The final interpretation of AKO depends on conservative horizon definition. The site is archaeologically complex, with a monumental early medieval phase overlying or intersecting Late Antique Christian contexts. The initial 53-sample series, therefore, had to be filtered. The decision to exclude uncertain C5-area individuals, retaining only three C5 individuals as Gothic-associated, reduces the sample size but increases interpretive reliability. It also avoids the risk that a later medieval, probably Pecheneg-period, horizon could be mistakenly used to define late antique Gothic ancestry.

READv2 identified close relatives among individuals connected to the suspected later-horizon group, and archaeological reassessment independently flagged the same burials as probably belonging to Pecheneg-period; the two lines of evidence converge. Within Bulgarian archaeology, genome-wide kinship analysis has not yet been a standard tool for adjudicating multi-phase cemetery contexts, and the C5 case illustrates how it can separate chronological horizons that stratigraphy and typology leave ambiguous. We, therefore, offer it as an instrument that is now available to Bulgarian archaeological practice.

The final 37-individual dataset should, therefore, be understood as the clean and conservative dataset for the Gothic-period analysis. The excluded individuals form the basis for a separate study of a probable Pecheneg-period horizon at AKO, which we plan to report. For the purposes of the present paper, their exclusion sharpens the demographic resolution of the late antique Gothic-associated record.

### 3.5 Kinship and community structure

The kinship results are consistent with local cemetery communities rather than an inter-site biological network. Close relatives are confined within sites, which indicates that Aquae Calidae and AKO were not simply two burial grounds of one sampled kin group. At the same time, the presence of family relationships within sites indicates that the cemeteries were not random military deposits or merely transient assemblages. They represent socially reproducing communities, or at least family-level burial clusters, embedded in different local settings under a shared Gothic-associated cultural horizon.

### 3.6 Limitations

Several limitations constrain the interpretation. First, sample sizes for individual phases are small, especially after conservative C5 filtering. The retained C5 set is reliable for inclusion but not sufficient for strong population-level claims about post-Nedao ancestry dynamics. Second, reference panels remain imperfect. No published Roman-era central Anatolian reference population fully matches the historical period; Kalehöyük and Iznik are proxies for ancestry streams, not literal source populations (Lazaridis et al., 2022; Olalde et al., 2023). Published Chernyakhov samples are derived from limited geographic contexts and may not represent the whole Chernyakhov–Sântana de Mureş range (Järve et al., 2019; Saag et al., 2025). Third, DATES estimates the admixture timing but not the place (Narasimhan et al., 2019). Finally, genetic heterogeneity is compatible with tradition-bearing cultural–political or frontier-formation frameworks, but it cannot prove the institutional mechanism through which Gothic identity was maintained (Wolfram et al., 1988; Pohl and Heydemann, 2013; Kulikowski, 2007).

### 3.7 Implications

The study contributes a Balkan dataset to the wider archaeogenomic record of the Migration Period and a model-frame approach for testing Gothic-associated material identity against demographic evidence. The results indicate that the appropriate null is not a search for “the Gothic genome” but rather the question of whether Gothic-associated cultural labels correspond to different combinations of northern/Pontic, Balkan, Anatolian/Roman-provincial, steppe-related, and local ancestry. In Bulgaria, the answer is consistent across analyses: the Gothic-associated horizon contains real northern/Pontic ancestry, but it is embedded in genetically heterogeneous communities whose ancestry histories differ by site and sub-group.

The result demonstrates that ancient DNA can constrain historically named identities without reducing them to biology (Halsall, 2007; Geary, 2002; Steinacher, 2017). The genomes show the demographic substrate; archaeology and history define the cultural and political frame. When the two are interpreted together, the Balkan Gothic data support a formation process rather than a single descent line: migration into the lower Danube, admixture in a multiethnic Chernyakhov/frontier world, incorporation of Balkan–Anatolian Roman-provincial substrates, and local reproduction within distinct cemetery communities. The methodological observation in §3.4—that READv2 kinship analysis can identify cross-horizon mixing at multi-phase cemeteries—extends to the broader problem of dating-controlled sampling at long-used sites.

## 4 Methods

### 4.1 Sample collection and inclusion criteria

Bone and dental samples were collected from archaeological excavations associated with Aquae Calidae and the Aul of Khan Omurtag. The initial screened series comprised 53 individuals from the two sites. The final analytical series comprises 37 individuals: 20 from Aquae Calidae and 17 from AKO. The reduction followed archaeological reassessment and genomic quality control, with particular attention to C5-area burials at AKO. Individuals suspected to belong to a later medieval, probable Pecheneg-period horizon were removed. Additional uncertain individuals were excluded after READv2 identified close relatives linking some questionable individuals to the later-horizon group. Only three C5 individuals were retained because they had the strongest combined archaeological and genomic support for attribution to the Late Antique Gothic-associated horizon. All inclusion/exclusion decisions and the per-sample rationale are documented in Supplementary Tables S1, S2.

### 4.2 Ancient DNA data generation

Wet laboratory work was performed in the ancient DNA laboratory at Harvard Medical School in Boston, United States. DNA extraction followed established silica-based protocols for highly degraded ancient DNA, using silica beads with Dabney buffer in a robotic workflow (Rohland et al., 2018). Libraries were prepared using two protocols depending on the sample quality. Most data were generated from double-stranded libraries with partial UDG treatment (n = 74 libraries from 37 individuals), following Rohland et al. (2015). A smaller number of single-stranded libraries with USER treatment were prepared for two individuals (n = 4 libraries), following Gansauge et al. (2020).

All libraries were enriched by in-solution capture targeting the Twist Ancient DNA panel, using Rohland et al. (2022) reagents. Double-stranded libraries were captured with Twist1.44Mv1.5 reagents, and single-stranded libraries were captured with Twist1.4Mv1.8 reagents. Double-stranded libraries were sequenced on an Illumina NovaSeq X 10B instrument, while single-stranded libraries were sequenced on an Illumina NovaSeq X 25B instrument. Library-level quality metrics, pass/fail assessments, and per-sample coverage statistics are reported in Supplementary Table S1.

### 4.3 Bioinformatic processing

The great majority of data derive from sequencing products of in-solution enrichment targeting more than one million known polymorphic sites. Libraries were marked with identification tags, including barcodes and indices, before pooled sequencing. Paired-end sequences were merged using overlap-consensus criteria, requiring no more than one mismatch in the overlap where the base quality was at least 20 or no more than three mismatches where base quality was below 20. Sequences that could not be merged under these criteria were not analysed. Adaptors and identification tags were removed using ADNA-Tools v2.1.0.

Merged sequences were aligned to the hg19 human reference genome with decoy sequences (hs37d5) using BWA SAMSE v0.7.15 with parameters -n 0.01 -o 2 -l 16500. Duplicate reads were marked using Picard MarkDuplicates v2.17.10. Merged sequences were also mapped to the mitochondrial DNA Reconstructed Sapiens Reference Sequence (RSRS) for mitochondrial-specific metrics. Bioinformatic processing produced key quality metrics, including authenticity estimates based on elevated terminal damage, contamination estimates, and endogenous content. Pseudo-haploid genotypes were generated by randomly sampling one allele at each covered targeted site.

### 4.4 PCA and f-statistics

PCA was computed with smartsnp v1.2.0 using scaling = “drift” (the SMARTPCA convention of Patterson et al., 2006) and projection of ancient individuals onto a modern West Eurasian reference space defined by 874 individuals across 60 HumanOrigins populations (Lazaridis et al., 2014; Lazaridis et al., 2016; modern panel composition listed in Supplementary Table S4; ancient comparator macro-groups in Supplementary Table S5; individual-level PC coordinates and subcluster assignments in Supplementary Table S2). Variance explained per principal component was computed using the full-trace denominator (sum of per-SNP variances across all retained variant SNPs), matching the convention used in published projected-ancient West Eurasian PCAs; in the final rerun used for Figure 2, PC1 = 0.84% and PC2 = 0.39% of the full-trace variance. The PC-axis orientation was fixed by reference to known modern population centroids so that reruns are visually comparable. Outgroup f_3_ and f_4_ statistics were computed in ADMIXTOOLS2 using block-jackknife standard errors (Patterson et al., 2012; Maier et al., 2023). f_3_ statistics measured shared drift between Gothic targets and reference populations using Old_Africa1 (the Allen Ancient DNA Resource composite African outgroup) as the outgroup. f_4_ statistics tested directional relationships among Chernyakhov, Balkan, and Anatolian-related references and evaluated whether the proposed qpAdm source relationships were formally supported.

### 4.5 qpAdm and right-population panel

qpAdm models were run in ADMIXTOOLS2. The fixed right-population panel comprises 11 populations chosen to span the major deep-ancestry axes relevant to West Eurasian source discrimination: Mbuti (African anchor); Kostenki14 and Villabruna (Upper Palaeolithic western Eurasia); Yana_UP and MA1 (Ancient North Eurasian/Siberian); DevilsCave and Karitiana (eastern non-African); and Natufian, Boncuklu_PPN, GanjDareh_N, and Iberomaurusian (pre-/early-Neolithic poles in the Levant, central Anatolia, the Zagros, and North Africa). Panel design follows the methodological logic of Harney et al. (2021), Williams et al. (2024), and Flegontova et al. (2025): the right set is informative because its members occupy differentially related positions relative to the candidate left-side sources, is older than the modelled Late Antique targets (temporal stratification), and is compact (n = 11) rather than maximalist—the latter to avoid the false-rejection inflation that occurs when oversized reference sets destabilise the covariance structure. This is closer to the conservative ancient reference framework of Lazaridis et al. (2022) than to a generic global outgroup panel; it retains the deep anchors of Lazaridis et al. (2016)’s O9 (Mbuti, Karitiana, Kostenki14, MA1) but replaces some present-day or generic eastern references with more ancient regional contrasts and adds early Southwest-Asian and North-African populations that are directly relevant to Mediterranean and Balkan modelling.

qpAdm was run in ADMIXTOOLS2 using a fixed right-population panel designed to distinguish West Eurasian deep ancestry streams while rotating historically relevant left/source proxies. Targets were defined in analysis-specific .ind files anchored to the final retained sample set. Population-level proximal models used Aquae Calidae PCA South (n = 11), Aquae Calidae PCA North (n = 8), the Aquae Calidae outlier I40570 analysed separately, AKO pooled excluding the low-coverage individual I40356 (n = 16), AKO C4 (n = 11), and AKO C5 (n = 3). Source populations were rotated across the Wielbark/Polish Iron Age, Chernyakhov-related, Balkan Iron Age/Late Antique, Anatolian/Marmara-related, Sarmatian/steppe-related, and distal deep-ancestry references. Models with p ≥ 0.05 and non-negative coefficients were treated as feasible; nested/popdrop models were reported where a full requested model was infeasible or a source was not required. The complete proximal qpAdm model set is reported in Supplementary Table S7. Individual-level qpAdm models, audited against the final retained sample set and interpreted descriptively, are reported in Supplementary Table S8. Distal qpAdm checks are reported in Supplementary Table S9 and Supplementary Figure S9. Proportions and standard errors are based on block-jackknife resampling. Population-level models exclude one individual from first-degree relative pairs where necessary to prevent family over-representation.

### 4.6 DATES and sensitivity controls

DATES v4010 (Narasimhan et al., 2019) was used to estimate admixture timing from ancestry covariance decay. Runs used binsize = 0.001, mindis = 0.02, maxdis = 0.5, qbin = 10, jackknife = YES, seed = 77, runfit = YES, afffit = YES, lovalfit = 0.6, minparentcount = 2, numchrom = 22, and a generation time of 29 years for calendar conversion (Moorjani et al., 2016). DATES was applied only after PCA, f-statistics, and qpAdm had identified plausible source contrasts. North-source and south-source sensitivity tests were used to evaluate whether the signal reflected a specific Wielbark-related contrast and whether southern proxies generated stable results. Specificity was tested by running DATES with the same north and south reference poles against non-Gothic Roman-period Balkan controls (Serbia_Viminacium_Roman_Rit and additional Roman-period Bulgarian populations); failure to recover the signal in those controls was treated as evidence for target-specific rather than regional structure. The complete configuration set, including failed and diagnostic runs and the full source-rotation matrix, is reported in Supplementary Table S10; the narrative sensitivity analysis is in Supplementary Note S6.

### 4.7 Uniparental markers and kinship

Y-chromosome haplogroups were assigned with yHaplo using diagnostic SNPs called at ≥ 3× coverage on terminal SNPs (ISOGG v15.73). Mitochondrial haplogroups were assigned with HaploGrep2 using PhyloTree Build 17 (Weissensteiner et al., 2016). READv2 (Monroy Kuhn et al., 2018) was used for kinship estimation with a kinship cutoff of 0.04 and a minimum SNP overlap of 15,000 per pair. READv2 returns kinship classifications (first, second, third degree) and does not identify phased haplotype tracts. Pairs with insufficient SNP overlap were flagged; first- and second-degree relationships were treated as reliable where coverage and SNP overlap were adequate. For population-level analyses, one individual from each first-degree pair was excluded to prevent family over-representation. READv2 also informed the horizon review for questionable C5-area individuals by identifying close relatives connected to suspected later-horizon samples (§§2.1, 3.4). The complete pair-level READv2 output, including cross-site pair status, is reported in Supplementary Table S3; the population-enrichment and temporal-distribution analyses are discussed in Supplementary Note S4.

## Data Availability

Raw sequencing data for individuals previously released as part of the wider dataset are available from the European Nucleotide Archive under accession PRJEB106907. BAM files for nine additional individuals deposited specifically for this study are available under accession PRJEB121210 (sample accessions ERS30837346-ERS30837354; run accessions ERR17599381-ERR17599389). Pseudo-haploid genotypes are available through Harvard Dataverse at https://doi.org/10.7910/DVN/7RVV9N. The final interactive PCA, f3, DATES and READv2 kinship visualisations are available in the project GitHub repository at https://github.com/StamovS/gothicadna-supplement.

## Supplementary material

SUPPLEMENTARY FIGURE S9 Distal qpAdm model results for the final retained Gothic-associated targets. Model definitions and full results are provided in Supplementary Table S9.

SUPPLEMENTARY TABLES S1–S10 Consolidated workbook containing sample metadata, analysis-set membership and inclusion/exclusion rationale, READv2 kinship results, PCA reference panels, ancient comparator definitions, outgroup f3 and f4 statistics, proximal and distal qpAdm models, individual-level qpAdm checks, and DATES outputs.

SUPPLEMENTARY NOTES S1–S6 Historiographic and archaeogenetic literature context; theoretical and historiographic framing; archaeological and historical context for Aquae Calidae and the Aul of Khan Omurtag; READv2 kinship analysis; interpretation of outgroup f3 affinity statistics; and DATES sensitivity analyses and source-rotation tests supporting the main article.
