# Supplementary material for: Paleogenomic evidence for genetic heterogeneity and prior admixture in Gothic-associated communities of late antique Bulgaria
Source: Front Genet. 2026 Jul 24;17:1864752. doi: 10.3389/fgene.2026.1864752 (PMC13446869; doi:10.3389/fgene.2026.1864752)
Supplement: Supplementary file 1 [file Supplementaryfile2.docx]

**Supplementary Notes S1–S6**

*Paleogenomic Evidence for Genetic Heterogeneity and Prior Admixture in Gothic-Associated Communities of Late Antique Bulgaria*

Stamov S, Chobanov T, Wang T, Stoeva K, Momchilov D, Aladzhov A, Chobanov K, Klasnakov M, Stamov G, Nikolov M, Nesheva D, Heather P, Toncheva DI, Zamfirov M, Lazaridis I, Reich D

*This document consolidates the six supplementary notes referenced in the main manuscript. Section numbering (S1.1, S1.2, …, S6.9) is preserved unchanged from the standalone notes so that cross-references in the main text remain valid.*

**Contents**

**Supplementary Note S1.** Gothic archaeogenetics: literature comparison from Stolarek 2019 to Golubiński 2026

**Supplementary Note S2.** Historiographic models of Gothic ethnogenesis: Wenskus, Wolfram, Pohl, Heather, Kulikowski

**Supplementary Note S3.** Archaeology and chronology of Aquae Calidae and the Aul of Khan Omurtag

**Supplementary Note S4.** READv2 kinship analysis: first- to third-degree relationships

**Supplementary Note S5.** Outgroup f3 statistics: analytical framework and comparator classes

**Supplementary Note S6.** DATES admixture dating: sensitivity analysis and source-rotation tests

*Each note carries its own reference list at the end of that note. Supplementary tables (S1–S10) and Supplementary Figure S9 are distributed separately.*

**Supplementary Note S1**

**Gothic archaeogenetics in context: from Stolarek 2019 to Golubiński 2026**

*A literature review for the interpretation of Gothic-associated populations in Late Antique Bulgaria*

Six recent archaeogenetic studies bear on the interpretation of the Bulgarian data presented in the main text: Stolarek et al. (2019), Järve et al. (2019), Stolarek et al. (2023), Olalde et al. (2023), Saag et al. (2025) and Golubiński et al. (2026). They are reviewed below in chronological order. The note identifies which published findings constrain the interpretation of Aquae Calidae and AKO, where those constraints are weaker than they are sometimes read, and which questions remain open in the published record.

**Table S1.1.** Published archaeogenetic studies referenced in this synthesis.

| **Study** | **Material / region** | **Main finding** |
| --- | --- | --- |
| Stolarek et al. 2019 | mtDNA from the Masłomęcz group, eastern Poland | High maternal-line diversity, with affinities to both Jutland / Kowalewko and Pontic-Caspian comparanda. |
| Järve et al. 2019 | Genome-wide data from steppe and post-Scythian / Chernyakhiv-associated individuals in Ukraine | Chernyakhiv-associated individuals overlap broadly with modern Europeans and show elevated Near Eastern-related ancestry relative to earlier local steppe groups; sample size limits resolution of internal structure. |
| Stolarek et al. 2023 | Genome-wide data from Iron Age Wielbark- and Medieval Slav-associated populations in Poland | Wielbark-associated Iron Age individuals modelled as a substantial northern / Scandinavian-related component admixed with local central and east-central European substrates. |
| Olalde et al. 2023 | Genome-wide transect of 1st-millennium CE Balkan individuals | Roman Imperial-era Anatolian-related ancestry across the Balkans, followed in later centuries by Central / Northern European and steppe-related contributions. |
| Saag et al. 2025 | Genome-wide North Pontic dataset including Chernyakhiv-associated individuals | Marked heterogeneity within the Chernyakhiv horizon, including both northern / central European-shifted and southern-shifted profiles. |
| Golubiński et al. 2026 | Genome-wide reanalysis of the Masłomęcz group | Predominantly Scandinavian-derived core with substantial incorporation of individuals carrying Baltic, Balkan, western / central European and Mediterranean-related ancestry. |

## Stolarek et al. 2019

Stolarek et al. (2019) reported mitochondrial DNA from the Masłomęcz group in eastern Poland. The assemblage showed substantial maternal-line diversity and contained haplogroups linking the group both to Jutland / Kowalewko and to Pontic-Caspian comparanda. The authors interpreted this pattern as compatible with contact and incorporation during the eastern or south-eastern expansion of Wielbark-associated groups rather than the transit of a closed founder population. Because the analysis was confined to mtDNA, it cannot resolve the autosomal contrast between Balkan-related and Anatolian-related components, which is the central interpretive axis for Aquae Calidae in the main text.

## Järve et al. 2019

Järve et al. (2019) generated genome-wide data from ancient steppe individuals in Ukraine, including a post-Scythian, Chernyakhiv-associated subset. The Chernyakhiv-associated individuals overlapped broadly with modern Europeans, lacked the East Asian-related component present in some earlier steppe contexts, and carried elevated Near Eastern-related ancestry relative to earlier local samples. The Chernyakhiv-associated sample size was small, and the study did not resolve internal structure within the Chernyakhiv horizon.

## Stolarek et al. 2023

Stolarek et al. (2023) analysed genome-wide data from Iron Age Wielbark-associated individuals and from Medieval Slav-associated populations in present-day Poland. The Wielbark-associated Iron Age series was modelled, using PCA, f-statistics, qpAdm and uniparental markers, as carrying a substantial northern or Scandinavian-related component admixed with central and east-central European substrates. The study did not recover a closed northern source: the northern component was embedded in a local admixture process. This is currently the strongest published genome-wide reference for a northern / Pontic component in Gothic-associated population history.

## Olalde et al. 2023

Olalde et al. (2023) reported genome-wide data from 1st-millennium CE individuals across the Balkans. The analysis documented Roman Imperial-period ancestry shifts involving Anatolian-related ancestry and, in later centuries, contributions from Central / Northern Europe and the steppe. The study did not target Gothic-associated assemblages, but it characterised the regional substrate against which any Late Antique Balkan cemetery population must be read. Anatolian-related ancestry was already present in Roman Balkan populations before the documented Gothic settlement south of the Danube.

## Saag et al. 2025

Saag et al. (2025) generated genome-wide data from individuals across a long chronological range in present-day Ukraine. The Iron Age Chernyakhiv-associated subset was internally heterogeneous: individual positions in the published PCA spanned both northern / central European-shifted and southern-shifted ranges, with some individuals approaching continental southern European reference variation. Relative to Järve et al. (2019), the study substantially expanded the published comparator base for the North Pontic Chernyakhiv horizon and showed that ‘Chernyakhiv-related’ is not a homogeneous population descriptor.

## Golubiński et al. 2026

Golubiński et al. (2026) analysed and reanalysed individuals from the Masłomęcz group of eastern Poland using PCA, outgroup f3, f4 homogeneity tests, individual qpAdm and kinship / ROH analyses. The community was characterised as carrying a predominantly Scandinavian-derived core together with incorporated individuals of Baltic, Balkan, western / central European and Mediterranean-related ancestry. The community-level interpretation was that a Gothic-associated archaeological assemblage need not be genetically homogeneous, even at a site treated in the literature as a Gothic core within the Wielbark horizon.

## Synthesis

Two patterns are stable across the six studies. First, every Gothic-associated assemblage analysed so far at genome-wide resolution contains substantial within-site ancestry diversity (Stolarek 2019, 2023; Saag 2025; Golubiński 2026), and the Roman-period Balkan substrate against which a Balkan Gothic-associated cemetery must be read is itself the product of mixed Mediterranean, Anatolian and Northern European inputs (Olalde 2023). Second, the strongest published genome-wide anchor for a northern / Pontic component in Gothic-associated population history is the Wielbark Iron Age series of Stolarek et al. (2023), and that anchor is itself embedded in a local admixture process rather than representing a closed source.

The Bulgarian material reported in the main text sits at the southern end of the geographic range covered by these studies. AKO is consistent with a northern / Pontic Gothic-associated pole comparable in kind to the Masłomęcz and Wielbark Iron Age assemblages, embedded in Balkan Late Antique ancestry. Aquae Calidae carries a southern Balkan / Anatolian-related component that is not captured by available Chernyakhiv references alone; it is compatible with the Roman Balkan substrate documented by Olalde et al. (2023) together with additional Anatolian-related contribution. The site-level contrast between AKO and Aquae Calidae is structurally different from the within-site heterogeneity reported for Masłomęcz: the two Bulgarian sites do not share the same source structure under qpAdm and do not share close kinship.

Three caveats follow from the literature comparison. First, genetic similarity to Wielbark, Chernyakhiv or Scandinavian-related references is evidence of ancestry affinity relative to the available reference panel and not evidence for ethnic identity. Second, an Anatolian-related component in a Late Antique Balkan cemetery is compatible with several distinct historical pathways — pre-existing Roman-provincial substrate, captivity, military service, ecclesiastical mobility — and the data presented here do not distinguish among them. Third, the Chernyakhiv reference base remains thin, and the heterogeneity reported by Saag et al. (2025) means that ‘Chernyakhiv-related’ is a proxy for a formation zone rather than a single population.

## References

Golubiński M, Baca M, Popović D, Speidel L, Schiffels S, Niezabitowska-Wiśniewska B, Kokowski A, Molak M. 2026. Cosmopolitanism in the depths of Barbaricum evidenced by archaeogenomic data from the Late Iron Age Goth community of the Masłomęcz group. Genome Biology 27:50. doi:10.1186/s13059-026-03969-4.

Järve M, Saag L, Scheib CL, Pathak AK, Montinaro F, Pagani L, et al. 2019. Shifts in the genetic landscape of the western Eurasian steppe associated with the beginning and end of the Scythian dominance. Current Biology 29:2430–2441.e10. doi:10.1016/j.cub.2019.06.019.

Olalde I, Carrión P, Mikić I, Rohrlach AB, Mallick S, Lazaridis I, et al. 2023. A genetic history of the Balkans from Roman frontier to Slavic migrations. Cell 186:5472–5485.e9. doi:10.1016/j.cell.2023.10.018.

Saag L, Utevska O, Zadnikov S, Shramko I, Gorbenko K, Bandrivskyi M, et al. 2025. North Pontic crossroads: Mobility in Ukraine from the Bronze Age to the early modern period. Science Advances 11:eadr0695. doi:10.1126/sciadv.adr0695.

Stolarek I, Handschuh L, Juras A, Nowaczewska W, Kóčka-Krenz H, Michałowski A, et al. 2019. Goth migration induced changes in the matrilineal genetic structure of the central-east European population. Scientific Reports 9:6737. doi:10.1038/s41598-019-43183-w.

Stolarek I, Handschuh L, Juras A, Nowaczewska W, Piontek J, Kozłowski P, Figlerowicz M. 2023. Genetic history of East-Central Europe in the first millennium CE. Genome Biology 24:173. doi:10.1186/s13059-023-03013-9.

**Supplementary Note S2**

**Historiographic models of Gothic origins and identity**

*Background for the interpretation of Gothic-associated archaeogenomic data from Late Antique Bulgaria*

This note summarises the historiography on which the three working models in §1.2 of the main text rest, and identifies the genomic expectations that follow from each. The argument is methodological. The customary sequence Scandinavia → lower Vistula / Wielbark → Chernyakhov–Sântana de Mureș → lower Danube and Balkans is a chain of distinct propositions; evidence for one link does not entail the others. The Bulgarian sites belong to late Roman and post-Hunnic frontier contexts in which migration, local incorporation, military recruitment, ecclesiastical organisation and Roman political categories could all have shaped who was buried in a Gothic-associated cemetery, and the historiography reviewed here makes that range of possibilities explicit.

## The migration chain

The first link, between Scandinavia and the lower Vistula / Wielbark horizon, is currently the strongest in archaeogenomic terms (Stolarek et al., 2023; Golubiński et al., 2026; Supplementary Note S1). A substantial northern or Scandinavian-related component in Wielbark-associated individuals is well supported, but ancestry affinity to a Scandinavian reference panel is not equivalent to descent from a single Gothic founder population, and cremation, social selection in burial, and the gap between archaeological culture and political identity preclude direct genetic confirmation of the Jordanes origin narrative (Jordanes, 1915; Goffart, 1988).

The second link, from Wielbark to Chernyakhov–Sântana de Mureș, remains historically and archaeologically a major model (Heather, 1996; Kokowski, 2007; Andrzejowski, 2019). Chernyakhov is, however, generally reconstructed as a polyethnic formation including Germanic-associated, Sarmatian and Alan steppe, and Dacian/Getic populations (Kulikowski, 2007; Halsall, 2007; Oltean, 2007). The published genome-wide data on Chernyakhov-associated individuals (Järve et al., 2019; Saag et al., 2025) are compatible with this reconstruction: the horizon is internally heterogeneous, and a single Chernyakhov genome is not recoverable from the present record. “From Chernyakhov” is therefore best read as a formation-zone designator rather than as a claim of direct descent.

The third link, from the North Pontic and lower Danubian world into the Balkans, is the most historically entangled. Gothic-associated groups entered the Roman frontier through multiple documented episodes — third-century raids and movements, the Tervingian and Ulfilan Christian context, the Danube crossings of 376 CE, the 382 CE foederati settlement, and post-Hunnic political reorganisations (Wolfram, 1988; Heather, 1991). These episodes need not have carried the same demographic profile. The appropriate question for ancient DNA is therefore not whether the Goths had a single genome-wide ancestry profile, but whether specific Gothic-associated cemeteries show shared or divergent ancestry streams, admixture dates and kinship structures.

## From continuous peoples to tradition-bearing formation

Earlier scholarship treated the Goths as a continuous East Germanic people within a wider framework of Germanic unity, projecting late-antique ethnic labels backward as stable collective units. The turning point in modern historiography is Reinhard Wenskus’s *Stammesbildung* (1961), which placed a tradition-bearing core (*Traditionskern*) at the centre of *gens* formation rather than racial or unchanging descent. Herwig Wolfram (1988) developed this approach for Gothic history; Walter Pohl (1998; Pohl & Heydemann, 2013) shifted the discussion from origin myths to processes of identification; and Patrick Geary (2002) examined how late-antique ethnic labels were subsequently retrofitted to support modern national narratives. The continuity of a *gens*, in this tradition, is carried by names, traditions, political leadership, law, military followings, religious frameworks and interaction with Rome, not by demographic identity in the modern national sense. The Vienna-school formulation is not an anti-migration model; it can accommodate movement, political expansion and the transmission of Gothic traditions, but it does not require that all individuals buried in Gothic-associated contexts share a single genome-wide ancestry profile.

## Three interpretive poles

Three positions in the historiography give distinct genomic predictions and together span the range of currently defended models.

*Wolfram and Pohl: tradition-bearing formation.* The Goths are treated as a *gens* whose identity was reproduced through leadership, tradition, law, religion and Roman interaction rather than as an unchanged descent group. The genomic prediction is that Gothic-associated contexts may carry inherited demographic signal from earlier Gothic or North Pontic horizons but need not share one ancestry profile across sites or phases; shared material culture and Christian burial practice can coexist with substantial site-specific heterogeneity.

*Heather: migrationist continuity with assimilation.* Heather (1991; 1996) gives greater weight to a socially broad Gothic community and to large-scale movement from the Baltic / Wielbark zone toward the Pontic world. The framework is not a purity model — migration plus assimilation fits it better than migration without assimilation — but it does predict a detectable northern / Pontic component in at least some Gothic-associated Balkan contexts, together with possible variation introduced by incorporation along the route and after settlement in Roman territory.

*Kulikowski: frontier formation.* Kulikowski (2007) is sceptical of using Jordanes as a frame for Gothic prehistory and emphasises the Roman frontier as the setting in which Gothic identity became historically visible and politically meaningful. The corresponding genomic prediction is strong local and site-specific heterogeneity, substantial Roman-provincial or Balkan ancestry, and weak or absent evidence for a single coherent northern source population underlying all Gothic-associated burials. The model does not deny movement; it denies that a Gothic archaeological or textual label identifies a single migrating descent group.

These three positions are not mutually exclusive. Halsall (2007) and Goffart (1988) provide additional methodological cautions against direct equations among material culture, political labels and stable ethnicity, and against treating Jordanes as transparent evidence for deep Gothic prehistory. The same cautions apply, with greater force, to inferences drawn from ancient DNA: genomic clustering can be over-interpreted as ethnicity, and archaeological labels can be over-interpreted as ancestry.

## Working models for the Bulgarian data

The three historiographic positions translate into the three non-exclusive working models tested in the main text. Their historical claims, genomic predictions and the analyses used to evaluate each are summarised in Table S2.1.

**Table S2.1.** Historiographic models and corresponding archaeogenomic expectations for the Bulgarian Gothic-associated data.

| **Model** | **Historical expectation** | **Genomic expectation** | **Relevant tests** |
| --- | --- | --- | --- |
| Migrationist continuity (Heather) | Substantial movement from northern / Pontic Gothic horizons into the Balkans. | Substantial Wielbark- or Chernyakhov-related component in at least some individuals or sites; possible similarity between sites. | PCA placement; outgroup f3; f4 statistics; qpAdm source attribution; DATES source specificity. |
| Tradition-bearing formation (Wenskus–Wolfram–Pohl) | Gothic affiliation maintained through traditions, law, leadership, religion and Roman political contexts across changing demographic substrates. | Shared cultural label may coexist with site-specific ancestry; some demographic continuity is possible, but no single stable genome-wide profile is required. | Comparison of ancestry, admixture timing and kinship under shared material and ritual attribution. |
| Frontier formation (Kulikowski) | Gothic affiliation produced, expanded or stabilised within Roman frontier conditions. | High heterogeneity; substantial local Balkan and Roman-provincial ancestry; weak single-source continuity from any one northern population. | Controls against local Roman Balkan populations; qpAdm and f-statistic tests of Balkan and Anatolian components; kinship structure. |

## Synthesis

The historiography does not prescribe a single model in advance. It identifies three positions that make different and partially overlapping demographic predictions and that are, in their current published forms, defensible on independent grounds. The Bulgarian data are read here against all three simultaneously, with the expectation that the best account combines elements of more than one model rather than vindicating any single one.

A conservative summary of the present historiographic and archaeogenetic record is therefore the following. A northern or Scandinavian-related component in Wielbark-associated populations is now well supported, but Chernyakhov was internally heterogeneous; Balkan Gothic-associated contexts should accordingly be expected to show variable combinations of northern / Pontic, local Balkan, Anatolian or Roman-provincial, and steppe-related ancestries. Throughout the main text we use “Gothic-associated” rather than “genetically Gothic”, treat ancestry profiles as demographic evidence rather than identity labels, and ask which historical models are compatible with the genetic data, rather than claiming that genetics can decide what Gothic identity “was”.

## References

Andrzejowski J. 2019. The Gothic migration through Eastern Poland: archaeological evidence. In: Cieśliński A, Kontny B, editors. Interacting Barbarians: Contacts, Exchange and Migrations in the First Millennium AD. Neue Studien zur Sachsenforschung 9. Warsaw / Braunschweig.

Geary PJ. 1983. Ethnic identity as a situational construct in the early Middle Ages. Mitteilungen der Anthropologischen Gesellschaft in Wien 113:15–26.

Geary PJ. 2002. The Myth of Nations: The Medieval Origins of Europe. Princeton University Press, Princeton.

Geary PJ. 2016. Austria, the writing of history, and the search for European identity. Austrian History Yearbook 47:1–11.

Goffart W. 1988. The Narrators of Barbarian History: Jordanes, Gregory of Tours, Bede, and Paul the Deacon. Princeton University Press, Princeton.

Golubiński M, Baca M, Popović D, Speidel L, Schiffels S, Niezabitowska-Wiśniewska B, Kokowski A, Molak M. 2026. Cosmopolitanism in the depths of Barbaricum evidenced by archaeogenomic data from the Late Iron Age Goth community of the Masłomęcz group. Genome Biology 27:50.

Halsall G. 2007. Barbarian Migrations and the Roman West, 376–568. Cambridge Medieval Textbooks. Cambridge University Press, Cambridge.

Heather P. 1991. Goths and Romans, 332–489. Clarendon Press, Oxford.

Heather P. 1996. The Goths. Blackwell, Oxford.

Järve M, Saag L, Scheib CL, Pathak AK, Montinaro F, Pagani L, et al. 2019. Shifts in the genetic landscape of the western Eurasian steppe associated with the beginning and end of the Scythian dominance. Current Biology 29:2430–2441.e10.

Jordanes. 1915. The Origin and Deeds of the Goths. Trans. C. C. Mierow. Princeton University Press, Princeton.

Kokowski A. 2007. Goci: od Skandzy do Campi Gothorum. Trio, Warsaw.

Kulikowski M. 2007. Rome’s Gothic Wars: From the Third Century to Alaric. Cambridge University Press, Cambridge.

Oltean IA. 2007. Dacia: Landscape, Colonisation, Romanisation. Routledge, London / New York.

Pohl W. 1998. Conceptions of ethnicity in early medieval studies. Archaeologia Polona 29:39–49.

Pohl W, Heydemann G, eds. 2013. Strategies of Identification: Ethnicity and Religion in Early Medieval Europe. Brepols, Turnhout.

Saag L, Utevska O, Zadnikov S, Shramko I, Gorbenko K, Bandrivskyi M, et al. 2025. North Pontic crossroads: Mobility in Ukraine from the Bronze Age to the early modern period. Science Advances 11:eadr0695.

Stolarek I, Handschuh L, Juras A, Nowaczewska W, Piontek J, Kozłowski P, Figlerowicz M. 2023. Genetic history of East-Central Europe in the first millennium CE. Genome Biology 24:173.

Wenskus R. 1961. Stammesbildung und Verfassung: Das Werden der frühmittelalterlichen gentes. Böhlau, Cologne / Graz.

Wolfram H. 1988. History of the Goths. Trans. T. J. Dunlap. University of California Press, Berkeley.

**Supplementary Note S3**

**Archaeological and historical context for Aquae Calidae and the Aul of Khan Omurtag**

This note provides archaeological and historical context for the two Gothic-associated sites analysed in the main text: the early Christian necropolis at Aquae Calidae (Akva Kalide) near Burgas in Thrace, and the Late Antique church-and-cemetery complex at the Aul of Khan Omurtag (AKO) near Han Krum / Shumen in Moesia Secunda. Figures in this note are numbered Supplementary Figures S3.1–S3.4, and the embedded tables S3.1–S3.3; these are distinct from Supplementary Table S3, the READv2 kinship workbook.

## S3.1 Historical setting

The sequence Scandinavia → lower Vistula / Wielbark → Chernyakhov–Sântana de Mureș → lower Danube and Balkans is a chain of historically linked but distinct propositions rather than a single demonstrated migration (see Supplementary Note S2). The Bulgarian sites sit at the southern end of this chain, in a Roman frontier and post-Hunnic context. The historical episodes relevant to their interpretation include third-century raids and movements across Moesia and Thrace; the 332 CE treaty between Constantine and the Tervingi; the Ulfilan settlement and the *Gothi minores* in Moesia (Heather 1991; Thompson 2008); the Danube crossings of 376 CE; the 382 CE foederati settlement; and the post-Nedao reorganisation following the collapse of Hunnic power in the mid-fifth century (Wolfram 1988; Heather 1996). These episodes need not have involved the same demographic profile.

**Supplementary Figure S3.1.** Schematic historical flowchart of Gothic-associated trajectories from southern Scandinavia and the Wielbark horizon through Chernyakhov, the lower Danube, and the fifth- and sixth-century Visigothic and Ostrogothic successor contexts.

## S3.2 Aquae Calidae: site, chronology, and attribution

Aquae Calidae lies near modern Burgas, close to Anchialos on the Black Sea coast. The sampled necropolis occupies part of the abandoned or reconfigured Roman bath complex: burials cut through or ignore earlier Roman bath architecture, indicating reuse of a monumental Roman setting as Christian funerary space. The cemetery is dated to the fourth century — most probably to its third quarter, and not later than *c.* 375 CE — on the basis of coins, ceramics, and associated grave goods (Momchilov and Klasnakov 2020).

The archaeological case for describing Aquae Calidae as Gothic-associated rests on a combination of indicators rather than on any single diagnostic artefact. These include east-west Christian burial orientation; ceramic vessels with lattice-like ornament comparable to material from other Bulgarian Gothic-associated contexts; cases of artificial cranial deformation; the use of pottery tiles in graves; glass beads with regional parallels; and a bone comb with a semicircular handle and concentric-circle ornament found close to the graves. The site lies in a region connected by Jordanes (1915) with Gothic raiding activity near the thermal springs of Anchialos in the later third century; the textual association is geographical rather than direct.

Because the excavated area covers only part of a larger burial ground, the social composition of the community cannot be reconstructed from archaeology alone. Plausible scenarios include a local mixed Christian community with Gothic associations, a group linked to frontier military service, refugees or descendants of populations connected with the Ulfilan movement south of the Danube, and descendants of mixed marriages or provincial captives incorporated into Gothic-associated communities. None is excluded by the published evidence.

**Supplementary Figure S3.2.** Archaeological plan of the early Christian necropolis at Aquae Calidae, Burgas. The burial area lies within the abandoned Roman bath complex; most graves are oriented east-west and document reuse of the bath structures as funerary space. After Momchilov and Klasnakov (2020, p. 35).

## S3.3 AKO / Han Krum: horizons and source base

The Aul of Khan Omurtag (AKO) near modern Han Krum / Shumen comprises at least two analytically distinct occupational horizons. The first is a Late Antique Christian burial-and-church complex with several churches and associated necropoleis. The second is an Early Medieval fortified Bulgarian residence traditionally linked to Omurtag’s building activity in the ninth century. The modern site name derives from the later Bulgarian monumental phase; the burials relevant to the present study belong to the Late Antique horizon.

The distinction matters for chronology. Published radiocarbon dates from the site concern the Early Medieval fortification system and associated settlement features rather than the Late Antique graves. Geophysical and excavation work has documented a rectangular double palisade line with radiocarbon determinations on animal bones from the palisade ditch falling broadly between the late seventh and ninth centuries (Henning et al. 2009; Balabanov and Stoeva 2015). These results inform the pre-stone or Omurtag-period fortification history; they do not date the fourth- to fifth-century Christian cemetery.

The Late Antique occupation is concentrated in the northern sector of the later aul and is marked by a cluster of churches and burial zones. In Bulgarian scholarship, particularly the work of Balabanov (2006, 2010), the complex has been interpreted as a Gothic episcopal centre; comparative and more recent syntheses have favoured a less committed description, as a Christian centre with Chernyakhov-, Gothic- or broader East Germanic-related cultural connections. Publication of the cemetery itself remains uneven: recent syntheses report 31 Late Antique graves across several sectors, but only a minority have a fully published inventory, and chronological relationships between sectors are not completely resolved.

**Supplementary Figure S3.3.** Site plan of the Aul of Khan Omurtag (AKO) near Han Krum / Shumen, showing the later fortified complex and the main Late Antique church areas. Adapted from Stoeva (2014).

## S3.4 The AKO churches and burial sectors

AKO includes multiple Christian buildings and burial zones, labelled in the present manuscript as churches N1–N5 and burial areas C1–C5. These labels are working designators used for internal cross-reference; they are not a fully resolved historical sequence and they do not encode named ethnic groups. The current archaeological understanding of each is summarised in Table S3.1.

**Table S3.1.** Late Antique and Early Medieval features at AKO referenced in the main manuscript.

| **Feature** | **Archaeological summary** | **Chronology** | **Notes** |
| --- | --- | --- | --- |
| Church N1 | Single-nave basilica with altar barrier, in the northern sector of the complex. Grave no. 6 in or near the narthex is the best-published fibula-bearing burial. | Later fourth to mid-fifth century, on the basis of architecture, coins, and associated finds. | Source of the strongest church-associated East Germanic dress evidence at the site. |
| Church N2 / N3 sector | Large basilica with an earlier church phase; rich graves east and south-east of the apse have yielded silver fibulae and other high-status objects. | Predominantly fifth century; some typological discussions extend into the early sixth century. | Elite Christian burials with East Germanic-associated costume elements. |
| Church N4 / Basilica 4 | Disturbed but important church area with rich burials, including fibulae, belt fittings, beads, and traces of high-status funerary treatment. | Fifth century, with the later end of that horizon most likely. | Later disturbance limits chronological precision. |
| Church N5 | Ninth-century Bulgarian basilica constructed over earlier burial areas. | Early Medieval, overlying Late Antique remains. | Documents long-term reuse; the Late Antique and Early Medieval horizons must be separated analytically. |
| Burial areas C1–C5 | Sequential burial areas used in the main manuscript to structure AKO sample context. | C1–C2 fall in earlier Late Antique horizons; C4–C5 require caution because of later disturbance and uncertain context for some burials. | Working structure used for genomic analysis; not a fully resolved historical sequence. |

## S3.5 The Gothic attribution at AKO

The strongest material evidence for a Gothic or broader East Germanic affiliation at AKO is dress-related. Comparative work on Late Antique ‘Germanic’ fibulae in Dacia Ripensis and Moesia Secunda places AKO among the more important south-Danubian findspots (Chakarov 2014). Paired fibulae in female dress positions, particularly in or near church-associated graves, are a more specific indicator than churches, pottery or coinage in isolation, and they support participation in an East Germanic dress koine commonly associated with Gothic groups south of the Danube (Bierbrauer 1994).

The key contexts are summarised in Table S3.2. Three are particularly informative: Church N1 grave no. 6, with paired silver double-plate fibulae; the Basilica N2 sector, with high-status silver fibulae from a burial east or south-east of the apse; and Basilica N4 grave no. 4, an east-west burial within the church containing fibulae, a belt buckle, a gilded brooch, beads and traces of a coffin.

The limitations of the attribution are equally relevant. Churches are Christian monuments and do not identify ethnic affiliation on their own. A synthronon or multiple basilicas indicate ecclesiastical status, but do not establish a Gothic bishopric in the historiographic sense. The fibulae and elite costume evidence support participation in an East Germanic-associated funerary and dress tradition for a subset of the burials. They do not, on the published evidence, characterise the population of the site as a whole.

**Table S3.2.** Late Antique AKO contexts most relevant to East Germanic or Gothic attribution.

| **Context** | **Evidence** | **Chronology** | **Strength of attribution** |
| --- | --- | --- | --- |
| Church N1, grave no. 6 | Pair of silver double-plate fibulae; female burial; lime layer beneath skeleton reported in later synthesis; burial in or near the narthex. | Later fourth to mid-fifth century horizon for Church N1. | Strong for an East Germanic-associated dress signal. |
| Basilica N2 sector | Pair of silver fibulae from a burial east or south-east of the apse; high-status grave goods. | Fifth century; some typological arguments extend into the early sixth century. | Strong, but context publication is incomplete. |
| Basilica N4, grave no. 4 | Two fibulae, belt buckle, gilded brooch, beads, coffin traces; east-west burial inside the church. | Fifth century, on typological grounds. | Strong for elite Late Antique Christian burial with East Germanic affiliations. |
| Basilica N5 sector | Child burial with glass, amber and clay beads; separate D-shaped buckle. | Approximately mid-fifth century for the bead assemblage. | Moderate; Christian and chronological significance clearer than ethnic specificity. |
| Site-wide cemetery | 31 Late Antique graves reported in recent synthesis; a minority with full published inventory. | Late fourth to fifth century; sixth-century continuity not securely demonstrated from published material. | Limited for ethnic attribution because most graves lack diagnostic finds. |

**Supplementary Figure S3.4.** Selected grave goods from high-status AKO burials, including gilded silver jewellery and fibulae from Late Antique church-centred graves. Images courtesy of the Archaeological Museum, Veliki Preslav.

## S3.6 Competing attributions

The published evidence is compatible with several overlapping interpretations of the Late Antique Christian phase at AKO. Table S3.3 sets out the four most commonly defended attributions and the degree to which each fits the published record.

**Table S3.3.** Competing archaeological interpretations of the Late Antique AKO horizon.

| **Attribution** | **Material emphasis** | **Burial rite** | **Chronology** | **Fit to AKO** |
| --- | --- | --- | --- | --- |
| Gothic / East Germanic Christian community | Paired double-plate fibulae; East Germanic female costume; elite jewellery. | East-west inhumation; burial in or near churches. | Predominantly late fourth to fifth century; some typological extension later. | Good for the fibula-rich female burials; partial for the cemetery as a whole. |
| Roman provincial Christian community with barbarian acculturation | Roman ceramics, infrastructure, amphorae; mixed dress assemblages. | Church burials with sparse inventory; Christian orientation. | Continuous third- to fifth-century Roman / Late Roman presence on site. | Good for the broader settlement context. |
| Mixed frontier or federate community | East Germanic dress combined with Christian burial and Roman provincial surroundings. | Civilian Christian burials rather than weapon-rich warrior graves. | Fifth-century frontier mobility in the Moesia Secunda / Thrace border zone. | A plausible synthesis of the published evidence. |
| Hunnic-period nomadic elite | Cranial modification; horse gear; weapon-rich graves; steppe prestige assemblages. | Predominantly non-Christian, non-church-centred. | Mid-fifth century if present. | Poor for the cemetery as a whole; at most consistent with isolated individuals or features. |

## S3.7 Implications for sample selection

The two sites are complementary in setting. Aquae Calidae is a coastal Thracian fourth-century necropolis in a region of historically attested Gothic movement; AKO is a longer-lived inland Christian complex in Moesia Secunda, within the lower-Danubian world of Ulfilas, the *Gothi minores*, federate politics and post-Hunnic reorganisation. Together they sample two demographic settings within the same broad cultural and religious horizon.

The archaeological record also constrains sample selection at AKO. The later medieval reuse of the site documented in §S3.3, and the unresolved context of some C5-area burials, mean that the published archaeological phase labels cannot be applied mechanically to genomic samples. In the main manuscript, individuals with uncertain or probable later-horizon assignment are excluded from the retained Gothic-period analytical set; the inclusion / exclusion rationale per individual is given in Supplementary Table S2.

## References

Antonova V, Dremsizova-Nelchinova Ts. 1981. Aulat na khan Omurtag pri Chatalar. DI “Septemvri”, Sofia.

Balabanov T. 2006. Gotski episkopski tsentar ot IV–VI v. do s. Han Krum, Shumensko. In: Goterna: Current Aspects of the Gothic Historical and Cultural Heritage in Bulgaria. Balkan Media, Sofia.

Balabanov T. 2010. Otnovo za gotite i gotskiya episkopski tsentar ot IV–V v. do s. Han Krum, Shumensko. In: Velikotarnovskiyat universitet “Sv. Sv. Kiril i Metodiy” i balgarskata arheologiya. Veliko Tarnovo.

Balabanov T, Stoeva K. 2015. Novi danni za ukrepitelnata sistema na Aula na khan Omurtag pri s. Han Krum, Shumensko, pp. 279–311.

Bierbrauer V. 1994. Archäologie und Geschichte der Goten vom 1.–7. Jahrhundert. Versuch einer Bilanz. Frühmittelalterliche Studien 28:51–171.

Chakarov K. 2014. Germanic fibulae from the cemeteries of the Late Antique provinces of Dacia Ripensis and Moesia Secunda. Proceedings of the Regional Museum of History, Veliko Tarnovo 28–29.

Dinchev V. 2024. Nekropolat kray kv. Benkovski, Sofia, i kasnoantichnite nekropoli v dneshna Balgariya. Book 1. Sofia.

Heather P. 1991. Goths and Romans, 332–489. Clarendon Press, Oxford.

Heather P. 1996. The Goths. Blackwell, Oxford.

Henning J. 2009. The metropolis of Pliska or, how large does an early medieval settlement have to be in order to be called a city? In: Henning J, editor. Byzantium, Pliska, and the Balkans. De Gruyter, Berlin / New York, pp. 209–240.

Henning J, Balabanov T, Milo P, Ziemann D. 2009. Khan Omurtag’s stone palace of AD 822: a ‘modernized’ eighth-century timber fort. In: Henning J, editor. Byzantium, Pliska, and the Balkans. De Gruyter, Berlin / New York, pp. 433–440.

Hristov M, Stoeva K. 2013. Sadove ot antichna pesh za bitova keramika do rannokhristiyanska tsarkva № 1 v aula do s. Han Krum, obshtina Preslav. Preslav 7:370–401.

Jordanes. 1915. The Origin and Deeds of the Goths. Trans. C. C. Mierow. Princeton University Press, Princeton.

Momchilov D, Klasnakov M. 2020. Rannokhristiyanski gotski nekropol Akva Kalide. In: Materiali ot Shestata natsionalna konferentsiya po istoriya, arheologiya i kulturen turizam “Patuvane kam Balgariya”. Shumen.

Stanev A. 2014. Observations on the barbaric presence in the province of Moesia Secunda in Late Antiquity.

Stoeva K. 2013. Grobna nakhodka ot vremeto na Velikoto preselenie na narodite ot rannokhristiyanska tsarkva № 1 v aula do s. Han Krum, obshtina Veliki Preslav. Preslav 7:354–369.

Stoeva K. 2014. Novootkrita tsarkva v aula do s. Han Krum. In: The Town in the Bulgarian Lands. Faber, Shumen.

Stoeva K. 2019. Severoiztochniya agal na palisadata v aula na kan Omurtag do s. Han Krum, obshtina Veliki Preslav.

Thompson EA. 2008. The Visigoths in the Time of Ulfila. 2nd ed. Duckworth, London.

Wolfram H. 1988. History of the Goths. Trans. T. J. Dunlap. 2nd ed. University of California Press, Berkeley.

**Supplementary Note S4**

**READv2 kinship analysis of Gothic-associated individuals**

This note reports the READv2 kinship analysis (Monroy Kuhn et al. 2018) of the final retained 37-individual Gothic-associated dataset (Aquae Calidae n = 20; AKO n = 17). The analysis uses the Relationship field of the workbook in Supplementary Table S3 — First Degree, Second Degree, or Third Degree — and is restricted to rows involving the final retained 37 individuals. An auxiliary Degree column in the workbook contains earlier or overlapping labels and is retained as metadata only. A 15,000-SNP overlap threshold distinguishes high-confidence relationship calls from low-overlap calls retained in the workbook for transparency but not used for inference here.

## S4.1 Data audit and evidence layers

The workbook contains 80 rows where the Relationship field is First, Second or Third Degree. Of these, 71 involve at least one individual in the final retained 37-set; the remaining 9 rows involve earlier or non-current Gothic-labelled IDs and are not used for the conclusions reported here. The 71 retained rows are partitioned by SNP overlap into a high-overlap layer (n = 42, ≥ 15,000 SNPs) used for inference and a low-overlap layer (n = 29, < 15,000 SNPs) retained as cautionary evidence. The high-overlap layer further partitions into 7 internal Gothic–Gothic rows and 35 Gothic-to-comparator rows. The full decomposition is in Table S4.1.

**Table S4.1.** Evidence layers in the READv2 workbook (Supplementary Table S3).

| **Layer** | **Rows** | **Description** |
| --- | --- | --- |
| First- to third-degree filtered rows in the workbook | 80 | All rows with Relationship = First Degree, Second Degree, or Third Degree, prior to anchoring on the final retained 37-individual membership. |
| Rows involving ≥ 1 final retained individual | 71 | Used for the manuscript-level conclusions in §2.8 of the main text. |
| Non-final retained Gothic-labelled rows | 9 | Rows involving earlier or non-current Gothic-labelled IDs absent from the final membership table; retained in the workbook audit only. |
| High-overlap rows (≥ 15,000 SNPs) | 42 | Main evidence layer; used for inference. |
| Low-overlap rows (< 15,000 SNPs) | 29 | Retained as cautionary evidence only; not used for close-relationship inference. |
| Of the 42 high-overlap rows: internal Gothic–Gothic | 7 | Within-site close kinship; see Tables S4.2 and S4.3. |
| Of the 42 high-overlap rows: Gothic–comparator external | 35 | All third-degree; see Tables S4.4 and S4.6. |
| Of the 42 high-overlap rows: Aquae Calidae–AKO cross-site | 0 | No cross-site close kinship detected at the high-overlap threshold. |

## S4.2 Internal kinship

All high-overlap first- to third-degree calls are internal to one site (Table S4.2). AKO contains six such pairs — one first-degree (I40356–I41199), two second-degree (I40356 with I41200 and I41206), and three third-degree (I41199–I41200, I41200–I40356, I41206–I40356) — involving a connected set of four individuals (I40356, I41199, I41200, I41206). The set constitutes a compact within-site cluster. Aquae Calidae contains a single retained internal pair, the third-degree call between I40572 and I40573 (θ = 0.050; 47,251 SNPs). No Aquae Calidae–AKO pair appears in the high-overlap layer at any degree.

I40356 carries the AKO low-coverage flag (Supplementary Table S2) and is excluded from population-level qpAdm and DATES modelling in the main text. Its high-overlap pair counts in Table S4.3 reflect unusually generous SNP overlap with three of the other AKO retained individuals (> 100,000 SNPs in each case) despite its overall coverage profile.

**Table S4.2.** High-overlap first- to third-degree pair counts in the final retained dataset (≥ 15,000 SNPs).

| **Context** | **First degree** | **Second degree** | **Third degree** | **Total** |
| --- | --- | --- | --- | --- |
| Within AKO | 1 | 2 | 3 | 6 |
| Within Aquae Calidae | 0 | 0 | 1 | 1 |
| Aquae Calidae – AKO cross-site | 0 | 0 | 0 | 0 |
| Gothic – external comparator | 0 | 0 | 35 | 35 |
| Total | 1 | 2 | 39 | 42 |

**Table S4.3.** Within-site high-overlap first- to third-degree relationships, ordered by relationship degree.

| **Site** | **Ind 1** | **Ind 2** | **Relationship** | **Kinship θ** | **Overlap SNPs** | **Note** |
| --- | --- | --- | --- | --- | --- | --- |
| AKO | I41199 | I40356 | First Degree | 0.209 | 103,977 | AKO kinship cluster; I40356 carries low-coverage flag and is excluded from population-level qpAdm and DATES. |
| AKO | I41200 | I41206 | Second Degree | 0.129 | 1,064,808 | AKO kinship cluster. |
| AKO | I41199 | I41206 | Second Degree | 0.097 | 1,067,402 | AKO kinship cluster. |
| AKO | I41199 | I41200 | Third Degree | 0.093 | 1,072,813 | AKO kinship cluster. |
| AKO | I41200 | I40356 | Third Degree | 0.091 | 103,836 | AKO kinship cluster; I40356 same as above. |
| AKO | I41206 | I40356 | Third Degree | 0.056 | 103,471 | AKO kinship cluster; I40356 same as above. |
| Aquae Calidae | I40572 | I40573 | Third Degree | 0.050 | 47,251 | Only retained Aquae Calidae internal pair. |

## S4.3 External comparators

All 35 high-overlap Gothic-to-comparator pairs are third-degree. No high-overlap first- or second-degree pair links a final retained individual to an external comparator. Apparent external first- and second-degree calls exist in the low-overlap layer (Table S4.5) but every such row falls well below the 15,000-SNP threshold; the overlap range is 300–3,145 SNPs, with the median below 1,500.

The external third-degree pattern is concentrated rather than dispersed (Table S4.4). Fifteen of the 35 high-overlap calls — 43% — involve a single comparator individual, DA189.SG from Russia_SaltovoMayaki.SG, paired with both AKO and Aquae Calidae individuals. The signal is panel-sensitive: it depends on the inclusion of one specific comparator and should not be read as evidence for direct Saltovo-Mayaki affinity at the population level. Three further calls involve Poland_Santok_IA.SG comparators and two involve Sweden_south_Pre_Viking.SG comparators; the remaining calls are singleton comparator-individual pairs. The full evidence layer is in Table S4.6.

**Table S4.4.** External comparator populations represented in the 35 high-overlap third-degree calls.

| **External comparator population** | **N** | **Gothic site(s)** | **External individual(s)** |
| --- | --- | --- | --- |
| Russia_SaltovoMayaki.SG | 15 | AKO; Aquae Calidae | DA189.SG (single comparator, repeated) |
| Poland_Santok_IA.SG | 3 | AKO; Aquae Calidae | PCA0394.SG; PCA0418.SG |
| Sweden_south_Pre_Viking.SG | 2 | Aquae Calidae | snb017.SG; snb018.SG |
| ChannelIslands_EIA.AG | 1 | AKO | I26630.AG |
| Czechia_EarlyMedieval_EarlySlav.SG | 1 | Aquae Calidae | RISE569.SG |
| Germany_EarlyMedieval_o1.SG | 1 | Aquae Calidae | NW54.SG |
| Germany_EarlyMedieval_o3.SG | 1 | AKO | AED513.SG |
| Hungary_Langobard_o1.AG | 1 | Aquae Calidae | SZ32.AG |
| Kazakhstan_Andronovo.SG | 1 | AKO | DA231.SG |
| Moldova_Glinoe_Scythian.SG | 1 | AKO | scy304.SG |
| Poland_Czarnowko_Wielbark_IA.SG | 1 | AKO | PCA0527.SG |
| Poland_Medieval_1.SG | 1 | Aquae Calidae | VK210.SG |
| Poland_Milicz_IA.SG | 1 | Aquae Calidae | PCA0514.SG |
| Poland_Poznan_IA.SG | 1 | Aquae Calidae | PCA0549.SG |
| Poland_PoznanSolacz_IA.SG | 1 | Aquae Calidae | PCA0554.SG |
| Poland_Viking.SG | 1 | Aquae Calidae | VK157.SG |
| Russia_Andronovo.SG | 1 | Aquae Calidae | RISE503.SG |
| Russia_Viking_o.SG | 1 | Aquae Calidae | VK413.SG |

**Table S4.5.** Low-overlap (< 15,000 SNPs) external first- and second-degree calls excluded from inference, sorted by SNP overlap.

| **Gothic ID** | **Site** | **External ID** | **External population** | **Relationship** | **Overlap** |
| --- | --- | --- | --- | --- | --- |
| I40929 | AKO | PCA0500.SG | Poland_PruszczGdanski_Wielbark_IA.SG | First Degree | 303 |
| I40929 | AKO | PCA0512.SG | Poland_Milicz_IA.SG | Second Degree | 300 |
| I40929 | AKO | PCA0424.SG | Poland_Santok_IA.SG | Second Degree | 355 |
| I40929 | AKO | PCA0418.SG | Poland_Santok_IA.SG | Second Degree | 432 |
| I40929 | AKO | VK200.SG | Poland_Viking.SG | Second Degree | 472 |
| I40929 | AKO | PCA0522.SG | Poland_PruszczGdanski_Wielbark_IA.SG | Second Degree | 559 |
| I40929 | AKO | PCA0352.SG | Poland_Dziekanowice_IA.SG | Second Degree | 615 |
| I40929 | AKO | PCA0090.SG | Poland_Maslomecz_Wielbark_IA.SG | Second Degree | 622 |
| I40929 | AKO | PCA0396.SG | Poland_Santok_IA.SG | Second Degree | 1,156 |
| I40573 | Aquae Calidae | PCA0380.SG | Poland_Santok_IA.SG | Second Degree | 1,621 |
| I40929 | AKO | PCA0487.SG | Poland_PruszczGdanski_Wielbark_IA.SG | Second Degree | 2,036 |
| I40929 | AKO | CGG021934 | SlovacRoman3CE | Second Degree | 2,084 |
| I40575 | Aquae Calidae | PCA0547.SG | Poland_Poznan_IA.SG | Second Degree | 2,252 |
| I40573 | Aquae Calidae | PCA0521.SG | Poland_Poznan_IA.SG | Second Degree | 2,347 |
| I40929 | AKO | PCA0334.SG | Poland_OstrowLednicki_IA.SG | Second Degree | 2,567 |
| I41148 | Aquae Calidae | PCA0554.SG | Poland_PoznanSolacz_IA.SG | Second Degree | 2,661 |
| I40929 | AKO | PCA0479.SG | Poland_PruszczGdanski_Wielbark_IA.SG | Second Degree | 2,813 |
| I40929 | AKO | AED513.SG | Germany_EarlyMedieval_o3.SG | Second Degree | 2,998 |
| I40356 | AKO | PCA0506.SG | Poland_Milicz_IA.SG | Second Degree | 3,145 |

**Table S4.6.** Full high-overlap external third-degree calls (≥ 15,000 SNPs).

| **Site** | **Gothic ID** | **External ID** | **External population** | **θ** | **Overlap SNPs** |
| --- | --- | --- | --- | --- | --- |
| AKO | I41203 | I26630.AG | ChannelIslands_EIA.AG | 0.048 | 17,835 |
| AKO | I40932 | AED513.SG | Germany_EarlyMedieval_o3.SG | 0.060 | 25,425 |
| AKO | I40356 | DA231.SG | Kazakhstan_Andronovo.SG | 0.048 | 47,918 |
| AKO | I40932 | scy304.SG | Moldova_Glinoe_Scythian.SG | 0.050 | 15,134 |
| AKO | I41201 | PCA0527.SG | Poland_Czarnowko_Wielbark_IA.SG | 0.049 | 16,298 |
| AKO | I41201 | PCA0418.SG | Poland_Santok_IA.SG | 0.049 | 18,883 |
| AKO | I41230 | PCA0418.SG | Poland_Santok_IA.SG | 0.057 | 19,638 |
| AKO | I41199 | DA189.SG | Russia_SaltovoMayaki.SG | 0.051 | 39,226 |
| AKO | I41200 | DA189.SG | Russia_SaltovoMayaki.SG | 0.056 | 39,165 |
| AKO | I41206 | DA189.SG | Russia_SaltovoMayaki.SG | 0.048 | 39,010 |
| AKO | I41230 | DA189.SG | Russia_SaltovoMayaki.SG | 0.051 | 39,314 |
| AKO | I41235 | DA189.SG | Russia_SaltovoMayaki.SG | 0.060 | 39,343 |
| Aquae Calidae | I41148 | RISE569.SG | Czechia_EarlyMedieval_EarlySlav.SG | 0.047 | 101,561 |
| Aquae Calidae | I41148 | NW54.SG | Germany_EarlyMedieval_o1.SG | 0.051 | 28,982 |
| Aquae Calidae | I41148 | SZ32.AG | Hungary_Langobard_o1.AG | 0.048 | 124,323 |
| Aquae Calidae | I41151 | VK210.SG | Poland_Medieval_1.SG | 0.048 | 50,921 |
| Aquae Calidae | I40572 | PCA0514.SG | Poland_Milicz_IA.SG | 0.057 | 18,262 |
| Aquae Calidae | I41136 | PCA0554.SG | Poland_PoznanSolacz_IA.SG | 0.048 | 18,605 |
| Aquae Calidae | I41148 | PCA0549.SG | Poland_Poznan_IA.SG | 0.047 | 16,149 |
| Aquae Calidae | I40572 | PCA0394.SG | Poland_Santok_IA.SG | 0.050 | 15,951 |
| Aquae Calidae | I41148 | VK157.SG | Poland_Viking.SG | 0.051 | 103,279 |
| Aquae Calidae | I40573 | RISE503.SG | Russia_Andronovo.SG | 0.049 | 50,516 |
| Aquae Calidae | I40571 | DA189.SG | Russia_SaltovoMayaki.SG | 0.064 | 28,746 |
| Aquae Calidae | I40572 | DA189.SG | Russia_SaltovoMayaki.SG | 0.067 | 21,380 |
| Aquae Calidae | I41134 | DA189.SG | Russia_SaltovoMayaki.SG | 0.052 | 38,766 |
| Aquae Calidae | I41135 | DA189.SG | Russia_SaltovoMayaki.SG | 0.050 | 39,201 |
| Aquae Calidae | I41139 | DA189.SG | Russia_SaltovoMayaki.SG | 0.047 | 39,242 |
| Aquae Calidae | I41146 | DA189.SG | Russia_SaltovoMayaki.SG | 0.051 | 39,217 |
| Aquae Calidae | I41147 | DA189.SG | Russia_SaltovoMayaki.SG | 0.066 | 25,401 |
| Aquae Calidae | I41150 | DA189.SG | Russia_SaltovoMayaki.SG | 0.052 | 39,089 |
| Aquae Calidae | I41193 | DA189.SG | Russia_SaltovoMayaki.SG | 0.053 | 39,219 |
| Aquae Calidae | I41195 | DA189.SG | Russia_SaltovoMayaki.SG | 0.049 | 39,112 |
| Aquae Calidae | I40571 | VK413.SG | Russia_Viking_o.SG | 0.055 | 23,533 |
| Aquae Calidae | I41148 | snb017.SG | Sweden_south_Pre_Viking.SG | 0.054 | 30,557 |
| Aquae Calidae | I41148 | snb018.SG | Sweden_south_Pre_Viking.SG | 0.057 | 92,317 |

## S4.4 Summary

Two points support the §2.8 discussion in the main text. First, the two Bulgarian sites do not form an extended close-kin network: no first-, second- or third-degree pair at the high-overlap threshold connects Aquae Calidae and AKO. Second, the two sites differ in internal kinship density: AKO contains a compact within-site cluster of four individuals connected by first- to third-degree pairs, whereas Aquae Calidae is represented by one internal third-degree pair. All high-overlap external calls are third-degree; the strongest external signal — eleven calls to DA189.SG — is single-comparator driven and panel-sensitive, and external third-degree calls are accordingly reported as exploratory context rather than as evidence for specific historical affiliation.

## References

Monroy Kuhn JM, Jakobsson M, Günther T. 2018. Estimating genetic kin relationships in prehistoric populations. PLOS ONE 13:e0195491.

**Supplementary Note S5**

**Outgroup f3 affinity statistics for Gothic-associated assemblages in Late Antique Bulgaria**

This note provides the interpretive framework for the outgroup f3 statistics reported in § 2.3 of the main text and the comparator structure used to read them. Full statistical output is in Supplementary Table S6.

## S5.1 Analytical framework

Outgroup f3 statistics of the form f3(Old_Africa1; Target, Reference) were computed in ADMIXTOOLS2 with block-jackknife standard errors (Patterson et al. 2012). Old_Africa1 is the composite African outgroup used in the Allen Ancient DNA Resource framework. Higher f3 values indicate greater shared genetic drift between the target and the reference, relative to the outgroup. In this study the principal targets are the retained Gothic-associated assemblages from Aquae Calidae and AKO. The statistics are descriptive affinity measures: they summarise broad similarity structure between targets and a defined set of references, and are not used here as source attributions or as evidence for migration routes.

Outgroup f3 ranks overall genome-wide similarity. A highly ranked reference may be close because of shared ancestry, similar ancestry-component mixtures, chronological proximity, or regional population structure; conversely, a historically relevant population need not rank highest if it is internally heterogeneous or captures only one component of an admixed target. Directional source attribution is evaluated separately through f4 statistics, qpAdm and DATES (§§ 2.4–2.6 of the main text).

## S5.2 Comparator classes

The reference populations were grouped into six broad comparator classes (Table S5.1) corresponding to the historical alternatives evaluated in the manuscript: northern and Pontic formation zones (Wielbark / Polish Iron Age and Chernyakhov-related); local and regional Balkan or Roman-period backgrounds; southern and eastern Mediterranean (Anatolian / Roman-Byzantine); steppe-sensitive references (Sarmatian and Scythian); broader Migration-period contextual groups; and East Asian-shifted steppe comparators retained as a control class. Raw population labels in Supplementary Table S6 follow the source-dataset conventions; the comparator-class labels used in this note (Wielbark / Polish Iron Age, Balkan / Roman-period, and so on) are chosen to avoid using ethnic terms as ancestry categories.

**Table S5.1.** Comparator classes used in the outgroup f3 analysis.

| **Comparator class** | **Illustrative references** | **Pattern in f3 results** | **Notes** |
| --- | --- | --- | --- |
| Wielbark / Polish Iron Age and Chernyakhov-related | Wielbark-associated Polish Iron Age groups; Chernyakhov-related references. | Higher relative affinity for AKO than for Aquae Calidae. | Tracks the northern / Pontic axis. |
| Balkan / Roman-period and Late Antique Balkan | Bulgaria Late Antiquity; Balkan Iron Age / Thracian; Serbia Roman-period. | Higher relative affinity for Aquae Calidae than for AKO. | Local and regional substrate within the lower Danubian and Balkan world. |
| Anatolian / Roman-Byzantine | Marmara / Iznik, Aegean / Anatolian, Roman-Byzantine. | Stronger relative affinity at Aquae Calidae than at AKO. | Southern and eastern Mediterranean axis; formal source adequacy is tested in §§ 2.4–2.5 of the main text. |
| Sarmatian- and Scythian-related | North Pontic and steppe-related Iron Age and Migration-period comparators. | Not dominant at the cemetery level; relevant to within-AKO individual-level shifts. | Steppe-sensitive control. |
| Migration-period contextual | Danubian, Central European, and other fifth- to sixth-century groups. | Often rank highly through shared broad European population structure. | Chronological context. |
| East Asian-shifted steppe | East Asian-shifted Hun or nomadic groups, where present. | Low affinity at the cemetery level for both sites. | Control for dominant East Asian-shifted input; does not test individual-level signal. |

## S5.3 Site-level f3 affinity patterns

The f3 results show the same site-level contrast observed in PCA, qpAdm and DATES. Aquae Calidae shows its strongest average affinities to Bulgaria Late Antiquity and related Balkan / Roman-period comparators, consistent with a southern Balkan, Roman-provincial or Anatolian-related component. AKO ranks these local Late Antique Balkan references lower, and shows relatively stronger affinity to Wielbark / Polish Iron Age, Chernyakhov-related, northern / Pontic, and broader Migration-period contextual references.

Many of the differences between high-ranking f3 values are small, often at the third or fourth decimal place. The contrast is therefore interpreted as a shift in relative affinity rather than as identification of any one population as a direct ancestor. The same directional difference recurs across PCA, outgroup f3 ranking, qpAdm source requirements, f4 statistics and DATES target behaviour; the convergence across these independent analytical layers, not the magnitude of any single f3 statistic, carries the result.

## S5.4 Geographic visualisation

Figure 3 in the main text visualises the f3 results geographically. Comparator populations are plotted at population-level coordinate centroids, where coordinates were available, and colour represents within-panel f3 rank: greener tones indicate higher f3 values relative to other mapped comparators for the same target, redder tones lower values. The figure displays affinity structure; it is not a migration map or a source-population assignment. Population labels are omitted from the map for legibility; full labels, f3 estimates, standard errors, Z-scores, ranks and mapped coordinates are in Supplementary Table S6.

## S5.5 Relation to formal modelling

The outgroup f3 results are complementary to, but less model-specific than, the formal ancestry analyses. They show that both sites participate in a broad northern / central European and Balkan affinity zone while also showing a site-level contrast: Aquae Calidae is shifted toward southern Balkan / Roman-period and Anatolian-related comparators, AKO toward northern / Pontic. Chernyakhov-related references are not always the top-ranked f3 comparators for either site; this reflects the internal heterogeneity of the published Chernyakhov sample (Järve et al. 2019; Saag et al. 2025), and Chernyakhov-related ancestry is evaluated as a source through f4 and qpAdm rather than from f3 ranking alone.

## S5.6 Limitations

Several limitations qualify the interpretation. (i) f3 values depend on the composition, date and internal heterogeneity of reference populations; reference choices that differ in any of these respects can shift rankings without reflecting a real change in target ancestry (Maier et al. 2023). (ii) Small differences among high-ranking populations are not individually informative. (iii) A low f3 rank for a historically relevant reference does not exclude ancestry related to that reference, particularly when the target is admixed. (iv) The geographic visualisation in Figure 3 uses population-level coordinate centroids and is intended for visual orientation only. (v) f3 cannot speak to the cultural, linguistic, legal or ecclesiastical mechanisms by which Gothic affiliation operated in Late Antique communities; those questions require integration with the archaeological and historical evidence (Supplementary Notes S2 and S3).

## References

Järve M, Saag L, Scheib CL, Pathak AK, Montinaro F, Pagani L, et al. 2019. Shifts in the genetic landscape of the western Eurasian steppe associated with the beginning and end of the Scythian dominance. Current Biology 29:2430–2441.e10.

Maier R, Flegontov P, Flegontova O, Işıldak U, Changmai P, Reich D. 2023. On the limits of fitting complex models of population history to f-statistics. eLife 12:e85492.

Patterson N, Moorjani P, Luo Y, Mallick S, Rohland N, Zhan Y, et al. 2012. Ancient admixture in human history. Genetics 192:1065–1093.

Saag L, Utevska O, Zadnikov S, Shramko I, Gorbenko K, Bandrivskyi M, et al. 2025. North Pontic crossroads: Mobility in Ukraine from the Bronze Age to the early modern period. Science Advances 11:eadr0695.

**Supplementary Note S6**

**DATES admixture dating: sensitivity analysis and source-rotation tests**

This note documents the DATES admixture-dating analyses summarised in § 2.6 of the main text. Figure 5 displays the weighted ancestry-covariance decay and source-rotation behaviour; Supplementary Table S10 contains the model-level numerical output. DATES is applied here after PCA, f-statistics and qpAdm have identified northern / Pontic and southern Balkan-Anatolian-related ancestry poles (Supplementary Notes S5 and the main-text §§ 2.3–2.5); it functions as a chronology test conditional on those poles, not as primary source attribution. Source labels (POL3 / Wielbark, Chernyakhov, BalkanCore, Mugla3, AnatoliaMax) are proxy pools used to create the allele-frequency contrasts that drive LD-decay dating (Narasimhan et al. 2019; Chintalapati et al. 2022) and do not denote literal source communities.

## S6.1 Targets

The DATES targets are analysis-specific labels with their own n values, distinct from the site-level retained-sample counts (Aquae Calidae n = 20; AKO n = 17; Supplementary Tables S1–S2). They are designed to maximise the north–south allele-frequency contrast for LD-decay dating and to mitigate single-target instability through subtarget pooling. The targets used in the runs reported here are summarised in Table S6.1.

**Table S6.1.** DATES targets and target-specific n values used in the source-rotation runs.

| **Target** | **n** | **Description** | **Role** |
| --- | --- | --- | --- |
| KalideN | 12 | Northern-shifted Aquae Calidae subtarget. | Primary Aquae Calidae timing target. |
| AKO | 11 | AKO subtarget for standalone runs. | Standalone AKO result, interpreted alongside qpAdm Chernyakhov-related modelling. |
| KalideN+AKO | 23 | Pooled KalideN and AKO subtarget. | Headline timing estimate. |
| KalideS | 5 | Southern-shifted Aquae Calidae subtarget. | Low-power heterogeneity control. |
| Roman Balkan controls | 3–16 | Serbia_Viminacium_RomanRit and Bulgaria_Roman_LA targets. | Specificity controls for whether the signal is generic to Roman Balkan structure. |

## S6.2 Source pools

The northern / Pontic source tests use Polish Iron Age / Wielbark-related references (POL3), broader POL3 + Scandinavian IA combinations, and a Chernyakhov-related proxy. The southern source tests use a BalkanCore pool, an Aegean Anatolian proxy from Muğla (Mugla3), and a broader AnatoliaMax pool. Union-south pools combine BalkanCore with Mugla3 or AnatoliaMax and are used as a stress test on the two-way DATES model.

**Table S6.2.** Source pools used in the reported DATES models.

| **Pool** | **Composition** | **Role** |
| --- | --- | --- |
| POL3 / Wielbark | Pruszcz Gdański, Masłomęcz and Kowalewko Wielbark / Polish Iron Age. | Primary northern proxy for KalideN and KalideN+AKO. |
| POL3 + Scandinavia_IA | POL3 expanded with Scandinavian Iron Age comparators. | Tests whether the contrast is specific to Polish IA / Wielbark or generic to northern European structure. |
| Chernyakhov | Chernyakhov-related proxy. | Intermediate Pontic proxy for AKO standalone runs. |
| BalkanCore | Balkan Iron Age and southern Balkan comparator pool. | Southern Balkan substrate proxy. |
| Mugla3 | Aegean Anatolian / Roman-Byzantine proxy from the Muğla region. | Aegean Anatolian proxy. |
| AnatoliaMax | Broader Anatolian-related pool. | Tests whether timing is stable when the southern source is widened across Anatolian variation. |
| Union-south | BalkanCore combined with Mugla3 or AnatoliaMax. | Two-way DATES model stress test. |

## S6.3 DATES settings

Runs used DATES v4010 with binsize = 0.001, mindis = 0.02, maxdis = 0.5, qbin = 10, jackknife = YES, seed = 77, runfit = YES, afffit = YES, lovalfit = 0.6, minparentcount = 2, numchrom = 22, and a generation time of 29 years. Calendar estimates were derived from burial-midpoint assumptions and are therefore approximate. Models with high Z, acceptable normalised root mean squared deviation (nrmsd) and stable behaviour under source rotation are reported as primary results; models with weak Z, inflated standard errors, negative point estimates or implausibly old timings appear in Supplementary Table S10 as model-failure diagnostics rather than as historical dates.

## S6.4 Primary results

The most informative single configuration is the pooled KalideN+AKO target with POL3 / Wielbark north and AnatoliaMax south: 12.44 ± 2.36 generations before burial (Z = 5.26; nrmsd = 0.186), a first-century CE point estimate with 95% CI ≈ 85 BCE – 183 CE. KalideN-only and pooled-target runs with BalkanCore, Mugla3 and AnatoliaMax converge within their standard errors, supporting an admixture signal in the range 11–15 generations before burial rather than a proxy-specific artefact.

**Table S6.3.** Principal DATES estimates reported in the main text and in Supplementary Table S10.

| **Target** | **North** | **South** | **Mean (gen)** | **SE** | **Z** | **nrmsd** | **95% CI** |
| --- | --- | --- | --- | --- | --- | --- | --- |
| KalideN+AKO | POL3 (Wielbark) | AnatoliaMax | 12.44 | 2.36 | 5.26 | 0.19 | 85 BCE – 183 CE |
| KalideN | POL3 (Wielbark) | Mugla3 | 12.74 | 2.70 | 4.73 | 0.19 | 136 BCE – 144 CE |
| KalideN+AKO | POL3 (Wielbark) | Mugla3 | 14.65 | 3.14 | 4.67 | 0.16 | — |
| KalideN | POL3 (Wielbark) | BalkanCore | 12.09 | 2.61 | 4.63 | 0.32 | 97 BCE – 179 CE |
| KalideN | POL3 (Wielbark) | AnatoliaMax | 11.01 | 2.42 | 4.55 | 0.24 | 97 BCE – 179 CE |
| KalideN+AKO | POL3 (Wielbark) | BalkanCore | 12.12 | 2.76 | 4.39 | 0.22 | 85 BCE – 183 CE |
| AKO | Chernyakhov | Mugla_Stratonikeia | 17.23 | 3.94 | 4.38 | 0.45 | 294 BCE – 214 CE |

## S6.5 North-source sensitivity

The DATES signal is strongest when the northern pole is anchored in POL3 / Wielbark-related references. Expanding the northern pool to include broader Scandinavian Iron Age comparators (POL3 + Scandinavia_IA) shifts point estimates older and inflates their standard errors across all three southern proxies (Table S6.4). The informative contrast is therefore not generic northern European ancestry but a Polish IA / Wielbark-related axis.

**Table S6.4.** Effect of expanding the northern source beyond POL3 / Wielbark.

| **Target** | **North** | **South** | **Mean (gen)** | **SE** | **Z** | **nrmsd** |
| --- | --- | --- | --- | --- | --- | --- |
| KalideN | POL3 (Wielbark) | BalkanCore | 12.09 | 2.61 | 4.63 | 0.32 |
| KalideN | POL3 + Scandinavia_IA | BalkanCore | 14.63 | 4.59 | 3.19 | 0.31 |
| KalideN | POL3 (Wielbark) | Mugla3 | 12.74 | 2.70 | 4.73 | 0.19 |
| KalideN | POL3 + Scandinavia_IA | Mugla3 | 17.26 | 4.63 | 3.73 | 0.17 |
| KalideN | POL3 (Wielbark) | AnatoliaMax | 11.01 | 2.42 | 4.55 | 0.24 |
| KalideN | POL3 + Scandinavia_IA | AnatoliaMax | 13.60 | 3.37 | 4.04 | 0.23 |

## S6.6 South-source rotation

BalkanCore, Mugla3 and AnatoliaMax produce timings that overlap within their standard errors for KalideN and KalideN+AKO. BalkanCore and AnatoliaMax give closely matched estimates for the pooled target (Table S6.5); Mugla3 gives a slightly older point estimate but remains within uncertainty. The pattern is consistent with a southern ancestry axis that was already structured or partly admixed before the sampled burial horizon, rather than with separately resolvable Balkan and Anatolian pulses. When southern proxies are pooled into broad union-south configurations, point estimates shift older and standard errors widen, as expected when two-way DATES assumptions are stressed by a heterogeneous southern source.

**Table S6.5.** South-source rotation for KalideN+AKO with POL3 / Wielbark north.

| **Target** | **South proxy** | **Mean (gen)** | **SE** | **Z** | **nrmsd** |
| --- | --- | --- | --- | --- | --- |
| KalideN+AKO | BalkanCore | 12.12 | 2.76 | 4.39 | 0.22 |
| KalideN+AKO | AnatoliaMax | 12.44 | 2.36 | 5.26 | 0.19 |
| KalideN+AKO | Mugla3 | 14.65 | 3.14 | 4.67 | 0.16 |

## S6.7 AKO, KalideS and Roman Balkan controls

AKO modelled alone produces a significant but less stable signal with Chernyakhov as the northern proxy and Mugla_Stratonikeia as the southern proxy: 17.23 ± 3.94 generations (Z = 4.38; nrmsd = 0.45). The lower stability is consistent with qpAdm modelling of AKO as Chernyakhov-related plus local Balkan / Late Antique ancestry: a partially admixed Chernyakhov proxy reduces the source contrast that DATES requires for crisp inference.

KalideS is a low-power heterogeneity control. Its reported configurations are weak or marginal and show only that the southern-shifted Aquae Calidae component does not produce the same crisp DATES behaviour as KalideN or KalideN+AKO. Non-Gothic Roman Balkan controls (Serbia_Viminacium_RomanRit and Bulgaria_Roman_LA) do not reproduce the high-Z ~12-generation signal under any of the source configurations tested. The DATES result is therefore specific to the Gothic-associated dataset rather than a generic feature of Roman Balkan population structure (Table S6.6).

**Table S6.6.** AKO, KalideS and Roman Balkan controls summarised from Supplementary Table S10.

| **Target** | **North** | **South** | **Mean (gen)** | **SE** | **Z** | **nrmsd** | **Status** |
| --- | --- | --- | --- | --- | --- | --- | --- |
| AKO | Chernyakhov | Mugla_Stratonikeia | 17.23 | 3.94 | 4.38 | 0.45 | 294 BCE – 214 CE |
| AKO | POL3 (Wielbark) | AnatoliaMax | — | — | 2.64 | — | weak |
| Serbia_Viminacium_RomanRit | POL3 (Wielbark) | BalkanCore | negative | — | < 1.8 | > 0.66 | model failure |
| Serbia_Viminacium_RomanRit | POL3 (Wielbark) | AnatoliaMax | negative | — | < 1.8 | > 0.66 | model failure |
| Serbia_Viminacium_RomanRit | BalkanCore | AnatoliaMax | negative | — | < 1.8 | > 0.66 | model failure |
| Bulgaria_Roman_LA | BalkanCore | AnatoliaMax | 130.8 | 57.7 | 2.27 | 0.34 | not interpretable |
| Bulgaria_Roman_LA | POL3 (Wielbark) | BalkanCore | 30.2 | 16.8 | 1.79 | 0.19 | wrong timeframe |
| Bulgaria_Roman_LA | POL3 (Wielbark) | AnatoliaMax | negative | — | < 0 | — | model failure |
| KalideS | POL3 (Wielbark) | BalkanCore | — | — | ~ 2.7 | — | insufficient contrast |
| KalideS | POL3 (Wielbark) | AnatoliaMax | — | — | ~ 3.2 | — | insufficient contrast |

## S6.8 Historical interpretation

The DATES results place the main north–south admixture signal well before the burial horizon. The 12.44 ± 2.36-generation headline estimate corresponds to an early Roman-period point estimate, with uncertainty extending from the late first millennium BCE into the second century CE. This timing precedes the documented fourth-century settlement episodes in the Balkans (Supplementary Note S3): it dates an ancestry mixture that had already occurred in the populations contributing to the Gothic-associated contexts at Aquae Calidae and AKO, not the formation of the burial communities themselves.

The DATES estimate complements rather than duplicates the other analyses. qpAdm identifies different source structures at Aquae Calidae and AKO; f4 statistics show that the Anatolian-related component cannot be explained by a Chernyakhov-only model; DATES constrains the timing of the relevant north–south mixture to a period before the sampled mortuary horizon.

## S6.9 Limitations

Several limitations qualify the interpretation. (i) DATES estimates are sensitive to source definition, target pooling and the allele-frequency contrast between source pools; the configurations reported here are not the only defensible ones. (ii) Point estimates do not identify named source populations: POL3 / Wielbark, Chernyakhov, BalkanCore, Mugla3 and AnatoliaMax are proxy constructs designed to drive LD-decay decomposition. (iii) Small sample sizes in some subtargets — particularly KalideS (n = 5) and standalone AKO (n = 11) — limit the precision of those runs. (iv) Intermediate proxies such as Chernyakhov are themselves partially admixed; this reduces source contrast and broadens standard errors. (v) Pooled targets may combine related but not identical historical mixtures, broadening the implied admixture-age distribution. Failed, negative or implausibly old estimates are retained in Supplementary Table S10 as model-failure diagnostics.

## References

Chintalapati M, Patterson N, Moorjani P. 2022. The spatiotemporal patterns of major human admixture events during the European Holocene. eLife 11:e77625.

Narasimhan VM, Patterson N, Moorjani P, Rohland N, Bernardos R, Mallick S, et al. 2019. The formation of human populations in South and Central Asia. Science 365:eaat7487.
